# Grafting Ink for Direct Writing: Solvation Activated Covalent Functionalization of Graphene

**DOI:** 10.1002/advs.202105017

**Published:** 2022-04-14

**Authors:** Yuanzhi Xia, Li Sun, Samuel Eyley, Brent Daelemans, Wim Thielemans, Johannes Seibel, Steven De Feyter

**Affiliations:** ^1^ Department of Chemistry Division of Molecular Imaging and Photonics KU Leuven Celestijnenlaan 200F Leuven B‐3001 Belgium; ^2^ Department of Chemical Engineering Sustainable Materials Lab KU Leuven Campus Kulak Kortrijk, E. Sabbelaan 53 Kortrijk 8500 Belgium

**Keywords:** covalent functionalization of graphene, grafting ink, solvation, electron transfer, direct writing, graphene patterning, reversible functionalization

## Abstract

Covalent functionalization of graphene (CFG) has shown attractive advantages in tuning the electronic, mechanical, optical, and thermal properties of graphene. However, facile, large‐scale, controllable, and highly efficient CFG remains challenging and often involves highly reactive and volatile compounds, requiring complex control of the reaction conditions. Here, a diazonium‐based grafting ink consisting of only two components, i.e., an aryl diazonium salt and the solvent dimethyl sulfoxide (DMSO) is presented. The efficient functionalization is attributed to the combination of the solvation of the diazonium cations by DMSO and *n*‐doping of graphene by DMSO, thereby promoting electron transfer (ET) from graphene to the diazonium cations, resulting in the generation of aryl radicals which subsequently react with the graphene. The grafting density of CFG is controlled by the reaction time and very high levels of functionalization, up to the failing of the Tuinstra–Koenig (T–K) relation, while the functionalization layer remains at monolayer height. The grafting ink, effective for days at room temperature, can be used at ambient conditions and renders the patterning CFG by direct writing as easy as writing on paper. In combination with thermal sample treatment, reversible functionalization is possible by subsequent writing/erasing cycles.

## Graphical abstract

A grafting ink displays unprecedented capability for the covalent functionalization of graphene, achieving a functionalized monolayer with ultrahigh grafting density. The writing is simple, efficient, and reversible, making the patterned covalent functionalization of graphene straightforward.

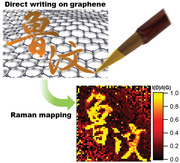

## 1 Introduction

Graphene as a typical 2D material consists of sp^2^ carbon atoms with its *π* electrons delocalized throughout the entire basal superlattice, resulting in unique electronic, mechanical, optical and thermal properties.^[^ ^1^, ^2^, ^3^, ^4^, ^5^, ^6^ ^]^ Tailoring these basic properties is crucial for the application of graphene‐based materials in a wide range of applications.^[^ ^7^, ^8^, ^9^, ^10^, ^11^ ^]^ A promising approach therefore is the covalent modification of the sp^2^ hybridized graphene lattice. However, due to the chemical inertness of the delocalized *π*‐system of graphene, highly reactive species are required to achieve a covalent functionalization of graphene (CFG).^[^ ^12^, ^13^ ^]^ Aryl radicals present such a reactive species, which has been extensively used for covalent modification of graphite and graphene. One of the approaches to generate aryl radicals is by the reduction of an aryl diazonium cation via electron transfer (ET) from graphene.^[^ ^14^, ^15^ ^]^ To this end, several strategies to promote the ET between graphene and diazonium cations to realize the CFG have been developed.

**Figure 1:**
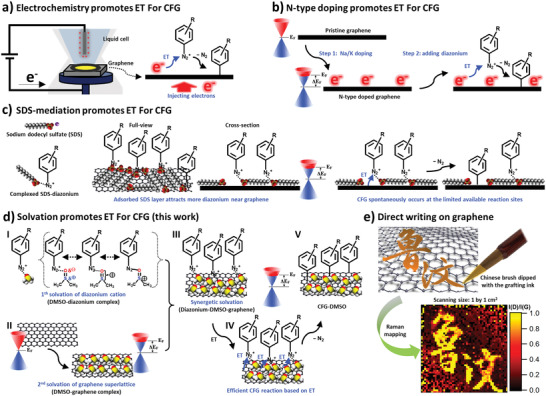
Approaches for the CFG based on the reduction of diazonium cations via ET from graphene. a) Electrochemistry promotes ET for the CFG. b) *N*‐type doping promotes ET for the CFG. c) SDS‐mediated ET for the CFG. d) Solvation promotes ET for the CFG: I) Solvation of diazonium by DMSO based on dipole–ion interactions; II) Solvation of the graphene lattice by DMSO based on dipole–induced dipole interactions; III–V) The grafting ink works at the interface where the diazonium‐DMSO‐graphene complex efficiently promotes the ET for the CFG; e) Direct writing on graphene: Raman map of I(D)/I(G) showing Chinese calligraphy. A soft Chinese brush was dipped with the grafting ink (80 × 10^−3^ m NBD) to write on graphene (1 by 1 cm^2^ SLG/SiO_2_). After drying of the sample in ambient conditions, Raman mapping revealed the Chinese name for Leuven.

Electrochemistry has been used to promote the ET and reduce the diazonium cations, whereby the graphitic substrate is used as the cathode in the electrochemical cell and thus injected with electrons (**Figure** **1**a).^[^ ^16^, ^17^, ^18^, ^19^, ^20^ ^]^ Although electrochemical grafting has been used extensively for the covalent functionalization of various surfaces, it is restricted to electrically conductive surfaces and the large‐scale surface functionalization remains challenging.

To achieve CFG without electrochemistry, Hirsch et al. developed a strategy for the diazonium‐based CFG by doping graphene with alkali metals. (Figure 1b).^[^ ^21^, ^22^, ^23^, ^24^, ^25^ ^]^ Alkali metals (Na/K) were shown to be effective *n*‐type dopants and were successfully used to tailor the charge carrier density of graphene.^[^ ^26^, ^27^, ^28^ ^]^ The doped graphene transfers electrons to diazonium cations, which are subsequently reduced to produce aryl radicals, which covalently attach to the graphene. While effective, this approach requires argon atmosphere, limiting its applicability under ambient conditions and potential for scale‐up. Recently, photoexcitation has been used to activate graphene and generate hot electrons that can reduce diazonium cations, resulting in the generation of aryl radicals and CFG. However, this protocol requires specific graphene samples and only a low grafting efficiency with limited control over the grafting layer could be achieved.^[^ ^29^ ^]^

In addition to the strategies based on activated reduction, Strano and co‐workers reported a series of studies on spontaneous ET to realize CFG.^[^ ^14^, ^15^, ^30^, ^31^, ^32^, ^33^, ^34^, ^35^ ^]^ Their experiments were performed by immersing a single layer graphene on SiO_2_ (SLG/SiO_2_) in aqueous solutions containing a diazonium salt and sodium dodecyl sulfate (SDS) at a controlled temperature (35–45 ℃) (Figure 1c). Thereby, the SDS plays a critical role in promoting the ET from graphene to the diazonium cation, i.e., the SDS acts as mediator by adsorbing on the graphene lattice with its hydrophobic alkyl chain. The negatively charged sulfate head group leads to *n*‐type doping of the graphene and tightly interacts with the diazonium cation, thus increasing its concentration near the graphene surface.^[^ ^34^, ^36^, ^37^, ^38^ ^]^ However, the SDS adsorption on the graphene is also a downside of this strategy as it limits the available reaction sites, resulting in a low degree of functionalization and long reaction times (7.0–16.5 h). Bypassing the ET chemistry of graphene, we recently developed a method by using reducing reagents (potassium iodide and ascorbic acid) to directly reduce diazonium salts to produce the aryl radicals for the CFG, thereby obtaining a relatively high degree of covalent functionalization.^[^ ^39^, ^40^ ^]^ While elegant, these chemical reduction‐based approaches require an additional reagent.

Despite the success of the aforementioned methodologies, strategies for facile, large‐scale, controllable and highly efficient CFG under ambient conditions are still an urgent need.^[^ ^41^, ^42^, ^43^ ^]^ Here, we present a facile method under ambient conditions for the covalent modification of graphene with a grafting ink, which solely consists of a solvent and an aryl diazonium salt (Figure 1d), rendering the patterning CFG by direct writing as easy as writing on paper (Figure 1e). Dimethyl sulfoxide (DMSO) was selected as the solvent (vide infra). Unprecedented high degrees of covalent functionalization are obtained, reaching levels where the Tuinstra–Koenig (T–K) relation,^[^ ^44^ ^]^ i.e., the I(D)/I(G) ratio in Raman measurements typically used to describe the degree of defects or functionalization of graphene, fails. The I(D)/I(G) ratios achieved with the aforementioned methods typically have values between 0.3 and 4.5 (Table S1, Supporting Information), while our approach leads to I(D)/I(G) values exceeding 5, where the T–K relation starts to fail. The degree of covalent functionalization can be controlled by adjusting the reaction conditions, i.e., reaction time and diazonium salt concentration. In contrast to many other approaches, the grafting layer remains at monolayer height (about 1.0 nm). Additionally, the addend group in the CFG can be adjusted by using different diazonium cations in the grafting ink, as demonstrated by using 4‐nitrobenzenediazonium (NBD), 4‐bromobenzenediazonium (BBD) and 3,5‐dichlorobenzenediazonium (DCBD). Notably, the grafting ink solution remains effective even after stored for 14 days at room temperature (RT), which is attributed to the stability of the DMSO‐diazonium complex.^[^ ^45^, ^46^, ^47^ ^]^ The graphene functionalization is reversible, i.e., pristine graphene can be restored by annealing to 400 °C and subsequently be functionalized again with the grafting ink.

## 2 Results and Discussion

### 2.1 Choosing the Solvent for the Grafting Ink

To select the solvent for the diazonium‐based grafting ink, several factors based on the solvent/diazonium cation and solvent/graphene interactions had to be considered. A sufficient solubility of the diazonium salts is required and the diazonium salt should be stable in the solution, i.e., not react with the solvent. For the solubility, a high solvent polarity is favorable. Some protic solvents, however, tend to react with diazonium cations, thus reducing the stability. For instance, water as protic polar solvent can dissolve diazonium salts, but the hydroxide anions present in water react with the diazonium cations, resulting in the formation of a precipitate after a few hours at room temperature.^[^ ^48^, ^49^ ^]^ In addition to the solvent/diazonium interactions, the solvent/graphene interactions have to be considered. That is, a strong affinity of the solvent to graphene is desirable to promote interactions between the diazonium molecules and graphene (vide infra). Further, the solvent has an impact on the electronic properties of graphene, i.e., a polar solvent can act as electron donor, resulting in the graphene to be *n*‐type doped,^[^ ^50^, ^51^, ^52^ ^]^ which can promote the electron transfer (ET) from graphene to the diazonium cation, thus promoting radical formation and subsequent CFG.

**Figure 2:**
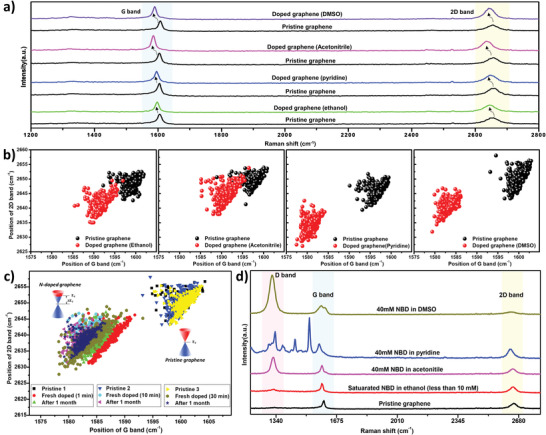
Screening the potential of different solvents for the grafting ink. a) Raman spectra of pristine SLG/SiO_2_ and after exposure to polar solvents (ethanol, acetonitrile, pyridine and DMSO). 100 µL solvent is drop‐casted on SLG/SiO_2_ for 10 min, followed by solvent removal and drying the doped SLG/SiO_2_ in an argon flow. b) The scatter plots of 2D band position versus G band position for the samples of the SLG/SiO_2_ before (pristine graphene) and after doping (doped graphene). c) The scatter plots of the samples of the SLG/SiO_2_ before and after doping by DMSO with different doping times, and the corresponding doped samples stored for 1 month under nitrogen atmosphere. d) Representative Raman spectra of the SLG/SiO_2_ functionalized by the grafting ink candidates (NBD dissolved with the candidate solvents). The corresponding Raman maps are shown in Figure S4 (Supporting Information). For the functionalization, 100 µL grafting ink is drop‐casted on pristine SLG/SiO_2_ for 10 min, followed by removal of the ink droplet and treatment in an argon flow.

Based on these criteria, we selected four candidate polar solvents for the grafting ink, i.e., ethanol, pyridine, acetonitrile, and DMSO. First, we determined the impact of the solvents on the electronic properties, i.e., doping of the graphene. Therefore, the pure solvents were drop‐casted on pristine SLG/SiO_2_ for 10 min. Subsequently, the solvent was removed and the graphene was dried under argon flow. To account for the spatial inhomogeneity of the graphene samples, Raman maps consisting of 400 single spectra (as 20 by 20 array) in an area of 50 × 50 µm^2^ were taken. The obtained Raman spectra (**Figure** **2**a) show a clear down‐shift (shift to a lower wavenumber) of the G band and 2D band after exposing pristine graphene to the solvents for 10 min, indicating *n*‐type doping.^[^ ^53^, ^54^ ^]^ Scatter plots of the 2D band versus G band positions (Figure 2b) further confirm the doping of the different graphene samples (see also Figure S1, Supporting Information). Thereby, DMSO and pyridine show similar degrees of *n*‐type doping, while ethanol and acetonitrile show a significantly lower degree of doping (Figure 2a,b). Considering the good *n*‐type doping of DMSO, combined with its low volatility, the time‐dependent DMSO‐doping of graphene (1, 10, and 30 min) was further investigated (Figure 2c and Figure S2, Supporting Information), demonstrating that a contact time of 10 min leads to saturation. Interestingly, the DMSO‐doped graphene retains its doping level when stored at RT under nitrogen for one month, indicating a strong adsorption of DMSO on graphene. Heating the sample to 400 °C results in un‐doped graphene (Figure S3, Supporting Information), indicating desorption of the solvent.

To confirm the potential of solvent‐mediated CFG, we drop‐casted NBD solutions with a concentration of 40 × 10^−3^ m onto SLG/SiO_2_ for 10 min, except for the ethanol solution (saturated concentration <10 × 10^−3^ m) due to the low solubility of NBD in the latter. Note that DMSO actually exhibits a very high solubility of NBD (>2.5 m), which is significantly higher compared to the other three solvents (<100 × 10^−3^ m).

We used Raman spectroscopy to confirm and characterize the CFG. The appearance of the D band in the Raman spectra of graphene indicates the existence of sp^3^ defects in the sp^2^ hybridized carbon lattice, which can be attributed to covalent functionalization. Thereby, NBD in DMSO showed the highest D‐band intensity, including a broadening of the G band caused by the emerging D′ band (vide infra), i.e., the highest degree of covalent functionalization of the screened solvents (Figure 2d). A smaller D band was observed with acetonitrile as solvent, while ethanol gave a very low D band and pyridine resulted in the formation of additional peaks, which are ascribed to side reactions of the dediazotization in pyridine.^[^ ^55^ ^]^ More details of the quantitative comparison of the I(D)/I(G) ratio are shown in Figure S4 (Supporting Information).

### 2.2 Optimization of the Grafting Ink for CFG

Having established that DMSO is the most promising solvent for a grafting ink, we further investigated the concentration and time dependency of the covalent grafting with NBD/DMSO solutions (grafting ink). The degree of grafting increases with increasing concentration of NBD under the fixed functionalization time of 1 min (Figure S5, Supporting Information), initially almost linearly up to a concentration of 80 × 10^−3^ m, then leveling off upon increasing the concentration further to 300 × 10^−3^ m. Therefore, we used a concentration of 80 × 10^−3^ m for the time‐dependent experiments.

**Figure 3:**
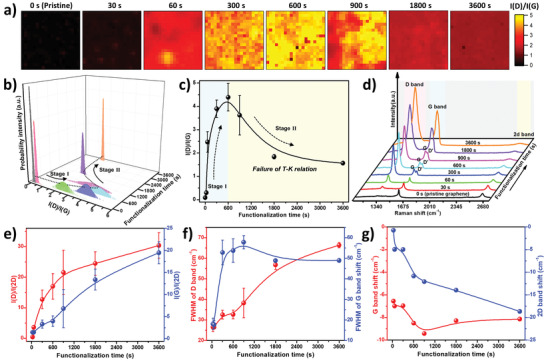
Time dependency of the covalent functionalization of SLG/SiO_2_ by the grafting ink (80 × 10^−3^ m NBD in DMSO). a) Raman maps showing the I(D)/I(G) ratio of the functionalized samples. b) I(D)/I(G) ratio distribution for the functionalized SLG/SiO_2_ as a function of functionalization time. c) Average I(D)/I(G) ratio based on the Raman maps versus functionalization time. d) Representative Raman spectra of the functionalized SLG/SiO_2_ with different functionalization times. e) Average I(D)/I(2D) and I(G)/I(2D) ratios versus functionalization time. f) FWHM of D and G bands versus functionalization time. g) Raman shift of G and 2D bands versus functionalization time. The average value and error bar (the standard deviation) are based on the 400 values from the Raman mapping (20 × 20, 50 × 50 µm^2^).

The Raman mappings in **Figure** **3**a show the evolution of the I(D)/I(G) ratio upon a stepwise increase of the functionalization time from 0 to 60 min. Initially, the I(D)/I(G) ratio increases with longer functionalization time and reaches its highest value (≈5) after 10 min (Figure S6, Supporting Information), but then decreases again below 2 after 60 min. To quantify the homogeneity of the grafted layer, the I(D)/I(G) ratio distribution for the corresponding Raman mappings was calculated (Figure 3b). The sharp distribution of the pristine graphene near zero shows that pristine SLG/SiO_2_ has a very low number of defects. For the functionalized graphene, the I(D)/I(G) ratio distributions initially broaden, up till a functionalization time of 10 min, and subsequently sharpen again for longer functionalization times. The initial broadening indicates a decrease in the homogeneity of the functionalized graphene, resulting from local variations of sp^3^ defects due to the covalent modification, thus indicating only partial modification of the graphene lattice. The sharpening obtained at longer functionalization times (>15 min, Figure 3b) reflects an increase in the homogeneity of the functionalized graphene. A similar trend was observed using ion bombarding to introduce the covalent defects in the graphene lattice.^[^ ^56^ ^]^

Plotting the absolute values of the I(D)/I(G) ratios versus the functionalization time shows initially an increase with a maximum around 4.4 after 10 min (Figure 3c). Counterintuitively, with longer reaction times (>10 min) the I(D)/I(G) ratio decreases again, which, according to the Tuinstra–Koenig (T–K) relation (vide infra) normally indicates a decrease in the degree of covalent modification. Note, however, that the Raman spectra show a further increase in the absolute D band intensity at longer reaction times (Figure 3d). Thus, the CFG process can be divided into two stages (stage I and stage II). After a functionalization time of 5 min a new band, i.e., the D′ band (Figure 3d), appears in the Raman spectra and shoulders the G band (stage I). At functionalization times above 10 min these two bands merge, leading to a big increase in the merged G band intensity. As a result, the I(D)/I(G) ratio decreases for longer functionalization times, even though the degree of covalent functionalization keeps increasing (stage II).

Typically, the density of defects in graphene is evaluated according to the T–K relation, which is the ratio of D‐ and G‐band intensity (I(D)/I(G) ratio).^[^ ^44^ ^]^ Thereby, a higher I(D)/I(G) ratio indicates a higher degree of covalent functionalization of the graphene lattice. However, the T–K relation has been shown to be valid only at relatively low defect densities and fails at high defect densities.^[^ ^56^, ^57^, ^58^, ^59^ ^]^ Thus, the T–K relation can only be used to describe the early stages of the CFG (stage I) and the Raman spectra evolve as follows: (i) D band appears and I(D)/I(G) ratio increases (stage I); (ii) D′ band appears; (iii) all bands broaden; (iv) G band and D′ band start to merge and form a single quasi merged G band (starting of stage II). In other words, the degree of CFG increases continually with increasing functionalization time, but can only be described with the T–K relation in stage I. In stage II, the T–K relation fails due to the high degree of CFG. Fortunately, the high degree of CFG can be objectively demonstrated by the continual increase of the I(D)/I(2D) and I(G)/I(2D) ratios with longer functionalization times (Figure 3e). Further, the increase in full width at half maximum (FWHM) of the D and G bands also unveil that the continued increase in the covalent defects density renders the broadening of the D and G bands (Figure 3f).^[^ ^57^ ^]^ Previously, a failing of the T–K relation due to the creation of a very high defect density has been clearly shown by bombardment of graphene with Ar^+^ or N^+^ ion beams.^[^ ^56^, ^57^, ^58^, ^59^ ^]^ Here, we demonstrated the breakdown of the T–K relation only based on the covalent modification graphene. Additionally, we were able to control the degree of CFG by varying the reaction time, and reach exceptionally high levels of functionalization. Other methods used for CFG generally only reach a degree of functionalization comparable to stage I (see the comparison in Table S1, Supporting Information). Note that the functionalized graphene remains *n‐*doped, i.e., DMSO solvent molecules remain adsorbed (Figure S7, Supporting Information). The degree of the *n*‐type doping is smaller compared to the graphene doped by pure DMSO, since the grafted nitrobenzene is an electron‐withdrawing group and has a *p*‐type doping effect. Consequently, the degree of *n*‐type doping reduces with increasing the amount of grafted molecules (Figure 3g).

**Figure 4:**
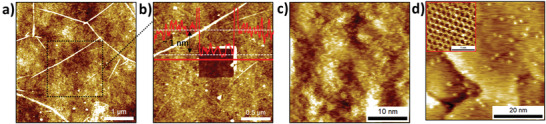
Layer morphology characterization using AFM and STM. a) AFM image of the functionalized SLG/SiO_2_ using the grafting ink (80 × 10^−3^ m NBD in DMSO) for 10 min. b) Amplified AFM image with a scratched area and line profile for layer height determination. c) STM image of the functionalized SLG/SiO_2_ using the grafting ink (80 × 10^−3^ m NBD) for 10 min. d) STM image of the functionalized SLG/Cu using the grafting ink (80 × 10^−3^ m NBD) for 10 min.

To further investigate the morphology of the grafted layer, the functionalized graphene was characterized by atomic force microscopy (AFM). Large‐scale AFM images show the formation of a uniform grafted layer on the functionalized graphene (80 mM NBD and 10 min) (**Figure** **4**a and Figure S8, Supporting Information). The thickness of the grafting layer was determined by partially removing the layer with the AFM tip in contact mode, revealing a layer thickness around 1 nm, which corresponds to monolayer formation (Figure 4b). Interestingly, increasing the reaction time from 10 to 30 min did not result in an increased layer thickness, i.e., the grafting reaction is limited to monolayer formation (Figure S8, Supporting Information).

To look into the grafting layer at the molecular scale, the functionalized SLG/SiO_2_ was imaged by scanning tunneling microscopy (STM), which is known to show grafted molecules as protrusions.^[^ ^20^ ^]^ In Figure 4c and Figure S9 (Supporting Information), STM images of the grafting layer on SLG/SiO_2_ confirm covalent functionalization, although the resolution is limited due to the intrinsic limitations of the sample, i.e., low electrical conductivity and high roughness.^[^ ^60^, ^61^, ^62^, ^63^ ^]^ The large‐scale images are dominated by the topographic features of the substrate SiO_2_ (Figure S9a, Supporting Information). The STM images obtained of the functionalized SLG/Cu clearly show the bright protrusions representing the grafted molecules (Figure 4d and Figure S9c, Supporting Information) due to the relatively good conductivity and local flatness of the substrate. Note that STM also allows the removal of the grafted molecules and imaging of the graphene lattice (inset of Figure 4d and Figure S9b,d, Supporting Information).

The high efficiency and the ease of use of the grafting ink allow direct writing on graphene. Inspired by the use of a soft roller for cleaning the surface of graphene,^[^ ^64^ ^]^ we used a soft Chinese brush, dipped with the grafting ink (80 × 10^−3^ m NBD in DMSO) for Chinese calligraphy (鲁汶 Chinese name of Leuven), on a 1 by 1 cm^2^ graphene sample (SLG/SiO_2_) (Figure 1e and Figure S10, Supporting Information). After drying in ambient conditions, Raman mapping was performed, whereby the I(D)/I(G) maps (Figure 1e) clearly show the successful Chinese calligraphy. Note that the average I(D)/I(G) of the writing area is not very high (≈1) compared to that in a bulk drop‐casting experiment. This may be attributed to the small volume of grafting ink used for optimal hand‐writing, or the influence of a reaction of the diazonium cations with the brush. In the future, nanopipette lithography could be used for efficient and complex CFG patterning at small scale.

### 2.3 Grafting Ink: Mechanism

The very high grafting density and the exclusive formation of monolayers can be understood by looking at the role of DMSO in the reaction mechanism. DMSO as polar solvent solvates the diazonium cation based on dipole–ion interactions, resulting in the formation of a DMSO‐diazonium complex and the high solubility of diazonium cations in DMSO.^[^ ^45^, ^46^, ^47^ ^]^ Additionally, DMSO has been shown to be a good solvent for stabilizing graphene in suspension. This is attributed to the formation of a stable DMSO solvation layer confined on the graphene lattice, in which the DMSO molecules in contact with the graphene lattice preferentially adopt either an oxygen‐down or oxygen‐up molecular orientation,^[^ ^65^, ^66^ ^]^ thereby affecting the electronic state of graphene.^[^ ^50^, ^51^, ^52^ ^]^ Likewise, we demonstrated *n*‐doping of DMSO, measured under dry conditions after removal of excess solution, indicating the strong affinity of DMSO to graphene and the formation of an adsorption layer, in agreement with previous reports.^[^ ^65^, ^66^ ^]^ Overall, DMSO has the capability to solvate the diazonium cation as well as to wet and dope graphene (Figure 1d). When the grafting ink is drop‐casted on graphene, we propose that solvation of the diazonium cation by DMSO and *n*‐doping of graphene by DMSO go hand‐in‐hand, thereby promoting an ET from graphene to the diazonium cations (Figure 1d III–V). Intermediate complex formation by solvation of reactants is known to dramatically promote reaction progress.^[^ ^67^, ^68^, ^69^ ^]^ Note that on highly oriented pyrolytic graphite (HOPG), which is known to be less reactive than graphene, the surface functionalization doesn't take place (Figure S11, Supporting Information). This may be attributed to the strong *π*–*π* interactions between the stacking layers preventing ET from the graphitic surface to the diazonium cation, as reported in previous studies.^[^ ^32^, ^70^, ^71^ ^]^

The monolayer formation further supports the hypothesis of ET from graphene to reduce the diazonium cation. That is, the adsorption of the diazonium cation on graphene is required for the ET to take place. The produced radical subsequently reacts with graphene and the first layer formed on graphene prevents additional ET. In contrast thereto, multilayer growth is typically observed in electrochemical reduction, where the ET can take place through the first layer or when the diazonium is chemically reduced in solution.^[^ ^20^ ^]^

### 2.4 Grafting Inks with Different Diazonium Salts

**Figure 5:**
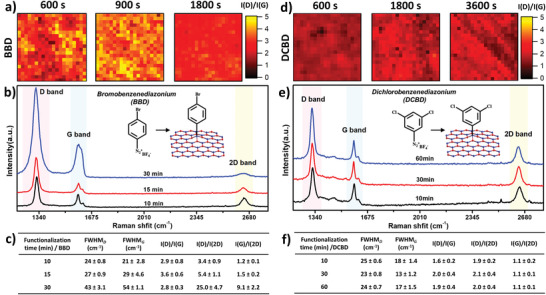
CFG by BBD and DCBD in DMSO, and the effect of functionalization time. a) Raman mappings, b) representative Raman spectra, and c) Raman spectra values of SLG/SiO_2_ functionalized with BBD grafting ink (80 × 10^−3^ m BBD in DMSO) for different functionalization times (10, 15, and 30 min). d) Raman mappings, e) representative Raman spectra, and f) Raman spectra values of SLG/SiO_2_ functionalized with DCBD grafting ink (80 × 10^−3^ m DCBD in DMSO) for different functionalization times (10, 30, and 60 min). The average value and error bar (the standard deviation) are based on the 400 values from the Raman mapping (20 × 20, 50 × 50 µm^2^).

To explore the versatility of the approach, we investigated the potential of other aryl diazonium cations in DMSO based grafting inks. Therefore, we prepared the grafting ink with 80 × 10^−3^ m 4‐bromobenzenediazonium salt (BBD) and 3,5‐dichlorobenzenediazonium salt (DCBD), respectively, and drop‐casted the grafting ink onto SLG/SiO_2_. Drop‐casting the BBD grafting ink resulted in an I(D)/I(G) ratio of 2.9 after a functionalization time of 10 min and 3.6 after 15 min (**Figure** **5**a–c). However, further increasing the functionalization to 30 min led to a decrease in the I(D)/I(G) ratio to 2.8, but with an increase in the absolute values of the D and G bands, thus indicating a very high degree of covalent functionalization, leading to the failure of the T‐K relation, as observed for the NBD grafting ink.

The I(D)/I(G) ratio (1.6) after a functionalization time of 10 min with the DCBD grafting ink is lower compared to the other diazonium salts, but nevertheless shows successful covalent modification (Figure 5d–f). Further increasing the functionalization time resulted only in a small increase in the I(D)/I(G) ratio to about 2 after 30 min. The difference in the efficiency of the covalent functionalization between aryl diazonium salts can be understood by taking into account the differences in chemical reactivity or electronegativity determined by their substituents,^[^ ^72^, ^73^ ^]^ although surface passivation by physisorption of by‐products cannot be excluded.

For chemical characterization of the functionalized samples, initially scanning electron microscopy/energy‐dispersive X‐ray spectroscopy (SEM‐EDS) measurements were carried out to visualize the functional surface and probe the element distributions. The SEM images (Figure S12, Supporting Information) show that the graphene surfaces functionalized with NBD, BBD, and DCBD have similar morphology to the pristine graphene. While EDS mapping has the potential to reveal the elemental composition of functionalized graphene surfaces,^[^ ^24^ ^]^ because of the extreme thin organic layer, and the limited sensitivity of EDS, signals are dominated by the underlying substrate (O and Si from SiO_2_/Si wafer) (Figure S13, Supporting Information). Subsequently, X‐ray photoelectron spectroscopy (XPS) measurements were performed. XPS measurements of the NBD grafted sample show the presence of the nitro group through the characteristic peak at 405.7 eV (Figure S14a, Supporting Information),^[^ ^74^ ^]^ while the BBD and DCBD samples show the presence of the corresponding C‐X species through characteristic signals for Br 3d_5/2_ at 70.3 eV^[^ ^75^ ^]^ and Cl 2p_3/2_ at 200.6 eV^[^ ^76^ ^]^ respectively (Figure S15a,b, Supporting Information). The carbon 1s spectra (Figures S14b and S15c, Supporting Information) of the grafted samples also show a shift in the peak carbon 1s binding energy associated with graphene (from 284.1 to 284.3–284.5 eV), along with the appearance of greater intensity on the higher binding energy side of the peak which can be attributed to C‐X states in the grafted samples.

To probe the stability of the grafting ink at RT, we characterized the covalent functionalization of SLG/SiO_2_ as well as SLG/Cu using grafting ink (80 × 10^−3^ m NBD in DMSO), which was stored for different times, i.e., freshly prepared or stored at RT for 3 days or 14 days, respectively (Figure S16, Supporting Information). On SLG/SiO_2_, the grafting efficiency decreases with a grafting ink stored for 3 days at RT, but still gives an I(D)/I(G) ratio of ≈4.1. Even the 14 days old grafting ink still gives an I(D)/I(G) ratio of ≈1.2 (Figure S16a,b, Supporting Information). Interestingly, the SLG/Cu samples showed a similar degree of functionalization with the 3 and 14 days old grafting ink, respectively, and only a small decrease in the I(D)/I(G) ratio (3.6 and 3.7) compared to that of the fresh grafting ink (4.5) (see in Figure S16c,d, Supporting Information). These results show that the grafting ink is still effective after being stored several weeks at RT, especially for modification of SLG/Cu. Nevertheless, the slow decrease in grafting efficiency suggests partial degradation or decomposition of the ink. By using ^1^H‐NMR and mass spectrometry (MS), the composition of the grafting ink (80 × 10^−3^ m NBD in DMSO) was followed over time at RT (see Figures S17–S19, Supporting Information). 4‐Nitrophenol is the main degradation product, in line with previous studies.^[^ ^46^, ^47^ ^]^ Additionally, some other byproducts emerge in low quantities at longer storage times at RT (Scheme S1 and Table S2, Supporting Information). After 14 days, the grafting ink solution still contains 59% of the original NBD content (Table S2, Supporting Information).

### 2.5 Thermal Stability of the Grafted Layer and Reversible CFG

**Figure 6:**
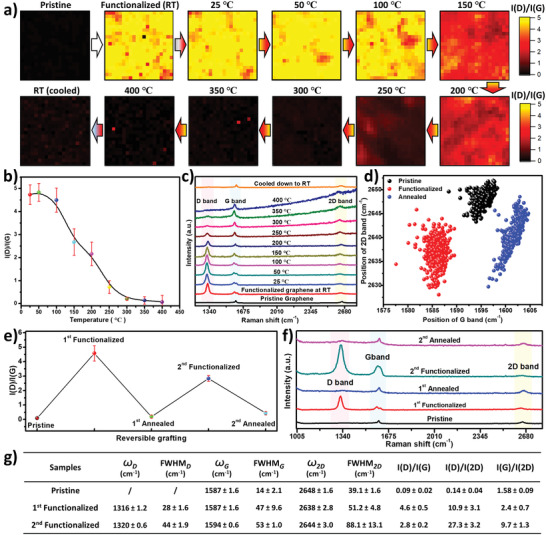
Thermal stability of the grafted SLG/SiO_2_ and reversible functionalization of SLG/SiO_2_ by the grafting ink (80 × 10^−3^ m NBD). Functionalization time is fixed at 10 min. a) Evolution of the Raman mapping of the functionalized SLG/SiO_2_ with a stepwise increase of the temperature from 25 to 400 °C. b) Average I(D)/I(G) ratios as a function of the annealing temperature. c) Representative Raman spectra of the functionalized SLG/SiO_2_ at different annealing temperatures. d) Scatter plots of 2D band versus G band positions for the same SLG/SiO_2_ sample in three states, pristine, functionalized (80 × 10^−3^ m NBD and 10 min) and de‐functionalized (annealed at 400 °C and cooled down to RT), respectively. e) Change of the average I(D)/I(G) ratios on SLG/SiO_2_ in response to functionalization/de‐functionalization cycles. f) Representative Raman spectra of the functionalization/de‐functionalization cycles. g) Raman spectra values of pristine, first‐time functionalized SLG/SiO_2_ and second‐time functionalized SLG/SiO_2_. The average value and error bar (the standard deviation) are based on the 400 values from the Raman mapping (20 × 20, 50 × 50 µm^2^).

The stability of the grafted layer on SLG/SiO_2_ was investigated by temperature dependent Raman spectroscopy. Therefore, the temperature of a grafted SLG/SiO_2_ sample was increased stepwise from 25 to 400 °C in a nitrogen atmosphere, while Raman mapping was used to monitor the changes in the I(D)/I(G) ratio at each temperature step (**Figure** **6**a–c). The I(D)/I(G) ratio does not change upon annealing to 50 °C and decreases only slightly at 100 °C. Further annealing above 100 °C results in a significant decrease in the I(D)/I(G) ratio at 150 °C and at 300 °C the I(D)/I(G) ratio reaches a level similar to the pristine sample. This evolution of the I(D)/I(G) ratio with increasing temperature indicates that a cleavage of the grafted molecules results in the restoration of the pristine graphene lattice upon annealing.^[^ ^77^ ^]^ Note that a small shift of the G and 2D bands in the Raman spectra of the restored graphene remains after annealing (Figure 6c,d), owing to the impact of thermal treatment on the state of the graphene lattice.^[^ ^78^, ^79^ ^]^

To confirm the restoration of the pristine graphene lattice upon annealing, we further explored the reversibility of the CFG using the grafting ink with the same concentration (80 × 10^−3^ m) and functionalization time (10 min). Therefore, a SLG/SiO_2_ sample was repeatedly functionalized and subsequently de‐functionalized by annealing to 400 °C, whereby a Raman mapping of the sample was performed after each treatment (Figure S20, Supporting Information). The average I(D)/I(G) ratios after annealing show that pristine graphene is mostly restored after the first as well as second functionalization cycle, while the average I(D)/I(G) ratio after the second functionalization is slightly lower compared to the first functionalization (Figure 6e). However, the individual spectra show broadening and higher bands after the second functionalization (Figure 6f,g). As discussed previously, this indicates a very high degree of functionalization, again leading to the failure of the T–K relation. This can be attributed to a state change of the graphene lattice after annealing, i.e., thermal treatment causes closer contact of the graphene lattice with the substrate, resulting in higher chemical reactivity.^[^ ^78^, ^79^ ^]^

The almost complete restoration of the graphene post‐annealing was also confirmed by XPS, where the nitrogen 1s spectrum (Figure S14a, Supporting Information) shows the disappearance of the characteristic nitro‐peak at 405.7 eV following annealing, and the shift of the carbon 1s peak (Figure S14b, Supporting Information) to a lower binding energy (284.0 eV). However, a slight intensity increase at the higher binding energy shoulder of the carbon 1s spectrum remains, along with an increased intensity in the nitrogen 1s spectrum at 400 eV when compared with the starting material, suggesting a low level of residual contamination.

## 3 Conclusion

In summary, we report a facile method for the chemical functionalization of graphene based on a grafting ink consisting only of the solvent DMSO and an aryl diazonium salt. The functionalization can be performed at ambient conditions and results in the formation of a grafted monolayer with a very high grafting density. Additionally, the functionalization is reversible and versatile, i.e., different aryl diazonium salts can be used. DMSO has been shown to be an efficient mediator in the covalent functionalization of graphene: its permanent dipole *n*‐dopes graphene as well as solvates the diazonium cation, promoting the ET from graphene for reduction of the aryl diazonium cation. Further, we demonstrate the potential to use the grafting ink for direct writing on graphene. A combination of this approach with inkjet‐printing would allow the direct and spatially selective functionalization of graphene or other 2D materials, which is the subject of an ongoing investigation. Additionally, this facile reaction pathway could open an avenue for the further exploration and utilization of polar organic solvents in promoting reactions involving ET chemistry of graphene.

## 4 Experimental Section

### Materials

4‐Nitrobenzenediazonium (NBD) tetrafluoroborate (98%) was purchased from TCI EUROPE. 4‐bromobenzenediazonium (BBD) tetrafluoroborate (96%), 3,5‐dichlorobenzenediazonium (DCBD) tetrafluoroborate, dimethyl sulfoxide (DMSO) (99.9%), and pyridine (99.8%)) were purchased from Sigma‐Aldrich. Ethanol (Spectroscopy purity) from EMD Millipore. Acetonitrile (Anhydrous, <0.001% water) from VWR CHEMICALS. All chemicals were used without further purification. High purity water (Milli‐Q, Millipore, 18.2 MΩ cm) was used for all experiments. The pristine samples of in situ CVD‐grown single layer graphene on copper (diameter 1 cm) (SLG/Cu) and the transferred single layer graphene to SiO_2_ (300 nm)/Si (1 × 1 cm^2^) (SLG/SiO_2_) were obtained from Graphenea and were used as received.

### Graphene Doping Experiments for Selecting the Ideal Solvent

Pristine samples of SLG/SiO_2_ were first characterized by Raman spectroscopy before the doping experiment. Then the same graphene samples were used to check the doping effect of the candidate solvents. Typically, 100 µL pure solvent was drop‐casted onto pristine SLG/SiO_2_ for 10 min, and then the graphene sample was dried under argon flow. The dried samples were stored in a nitrogen gas atmosphere prior to further characterizations.

### Covalent Functionalization of Graphene (CFG) by the Grafting Ink

Pristine samples (SLG/SiO_2_) were first analyzed for quality by Raman spectroscopy. NBD solutions were prepared by dissolving NBD in the 4 candidate solvents (DMSO, ethanol, pyridine and acetonitrile), and subsequently the effect of spontaneous CFG on SLG/SiO_2_ was tested for the 4 different NBD solutions. Typically, 100 µL NBD solution was drop‐casted onto the surface of SLG/SiO_2_ at room temperature (RT). After a given time, the CFG reaction was stopped by removing the droplet of NBD solution and rinsing the surface with the solvent used. The functionalized graphene was dried using argon flow. For DMSO, a series of CFG experiments were carried out probing concentration dependency, functionalization time dependency and reactivity of different diazonium cations (BBD and DCBD). In order to test the stability or lifetime of the grafting ink, the inks were stored at RT for different times, prior to using them for graphene functionalization. In addition, SLG/Cu was also used to repeat the CFG experiments. The dried samples were stored in a nitrogen gas atmosphere box prior to further characterizations.

### Raman Measurements

Raman characterization was performed at room temperature using a Raman microscope (Monovista CRS+, S&I GmbH). The 632.8 nm He‐Ne laser beam was directed and focused onto the surface of graphene (590 kW cm^−2^) using an objective (OLYMPUS, BX43 100×, N.A. 0.7). The Raman scattering signal was collected with the same objective and directed to a Raman spectrograph (Horiba JY, iHR‐320) equipped with a cooled charge‐coupled device (CCD) camera operating at −100 °C (Andor, DU920P) through a dichroic mirror, pinhole and a long pass filter (Chroma, HQ645LP). The accumulation time for a spectrum was 1 s. Raman mapping was performed based on array scanning (20 × 20, scan size 50 µm × 50 µm) resulting in 400 Raman spectra.

### Reversible CFG

Temperature‐dependent Raman measurements were performed in a Linkam stage HFS600, equipped with a liquid nitrogen controller for temperature control under a constant flow of nitrogen. A sample of functionalized SLG/SiO_2_ was set in the Linkam stage. Raman spectra were recorded at 25, 50, 100, 150, 200, 250, 300, 350, and 400 °C (heating rate is 150 °C min^−1^). Raman mapping was performed based on array scanning (20 × 20, scan size 50 µm × 50 µm). Pristine graphene was obtained upon heating to 400 °C. After cooling to room temperature, the sample was again exposed to the grafting ink leading again to CFG.

### AFM and STM Characterization

AFM images were obtained using the Cypher ES (Asylum Research) system at 32 °C in tapping mode at the air/solid interface. OMCL‐AC160TS‐R3 probes (spring constant≈26 N m^−1^) with a resonance frequency around 100 kHz were used for the AFM imaging. OMCL‐AC240TS‐R3 probes (spring constant ≈2 N m^−1^) with a resonance frequency around 70 kHz were used in the contact mode for locally removing, i.e., nanoshaving, the functionalized layer. All STM experiments were performed at room temperature (21–23 °C) using a PicoLE (Keysight) machine operating in constant‐current mode. STM tips were prepared by mechanical cutting of Pt/Ir wire (80%/20%, diameter 0.25 mm).

### SEM‐EDS Measurements

Scanning electron microscopy/energy‐dispersive X‐ray spectroscopy (SEM‐EDS) measurements were obtained using a FEI Quanta FEG‐250 environmental SEM equipped with a spectrometer for EDS. The SEM images were recorded with operating voltage of 30 kV and the EDS mapping was recorded with operating voltage of 5 kV.

### XPS Measurements

X‐ray photoelectron spectroscopy (XPS) characterization was performed on a Kratos Axis Supra photoelectron spectrometer with a monochromated Al K_*α*_ X‐ray source (120 W). Spectra were acquired using hybrid (magnetic/electrostatic) lens mode and slot aperture, with the analyzer operating in fixed analyzer transmission mode. Survey spectra were acquired with a pass energy of 160 eV, while high resolution spectra were acquired at 20 eV. Samples were electrically connected to the spectrometer using silver paint and measurements were made on three separate areas per sample. The binding energy scale was corrected to Ag 3d_5/2_ at 368.21 eV as measured on a clean silver foil on the same day as the sample analyses, using the same measurement conditions.

### NMR Measurements

Proton nuclear magnetic resonance (^1^H‐NMR) spectroscopy was performed on a Bruker AMX 400 MHz NMR spectrometer. The solution of 80 × 10^−3^ m NBD in DMSO‐d_6_ was prepared and kept at room temperature. Chemical shifts (*δ*) are reported in parts per million (ppm) referenced to tetramethylsilane (1H). 1 mg of dimethylsulfone (10.6 µmol) was added to 0.5 mL of the stock solution and was used as standard to calculate the amount of product present in the solution. The spectra were analyzed with MestReNova software.

### MS Measurements

Mass spectrometry (MS) characterization was performed on a RADIAN ASAP MS (Waters) with a single quadrupole mass detector using atmospheric pressure chemical ionization. The spectra were analyzed with MassLynx MS software.

### Statistical Analysis

The AFM images, STM images, and the corresponding 3D representations were analyzed using Scanning Probe Imaging Processor (SPIP 6.3.5) software from Image Metrology ApS. The Raman result analysis is based on the Raman mapping (20 × 20, scan size 50 µm × 50 µm). The D band (related to the presence of sp^3^ covalent defects), D′ band (sp^3^ covalent defects at high surface coverage), G band (intrinsic band to graphene), and 2D band (intrinsic band to graphene) were focused. The band position, intensity, and full width at half maximum (FWHM) of these bands were obtained. The intensity ratio of two different bands, such as I(D)/I(G), I(G)/I(2D) and I(D)/I(2D), were calculated t. Based on 400 values for every Raman map, further the statistical analysis regarding the band position, FWHM, and intensity ratio was done, obtaining the average value, error bar (the standard deviation), and histogram distribution.

## Conflict of Interest

The authors declare no conflict of interest.

## Supporting information

Supporting Information

## Data Availability

The data that support the findings of this study are available from the corresponding author upon reasonable request.
