# Supplementary material for: Grafting Ink for Direct Writing: Solvation Activated Covalent Functionalization of Graphene
Source: Adv Sci (Weinh). 2022 Apr 14;9(19):2105017. doi: 10.1002/advs.202105017 (PMC9259721; doi:10.1002/advs.202105017)
Supplement: Supplementary file 1 — Supporting Information [file ADVS-9-2105017-s001.pdf]

Supporting Information

Grafting Ink for Direct Writing: Solvation Activated Covalent Functionalization of Graphene

Yuanzhi Xia, Li Sun, Samuel Eyley, Brent Daelemans, Wim Thielemans, Johannes Seibel and Steven De Feyter**

Y.Z. Xia, L. Sun, B. Daelemans, J. Seibel and S. De Feyter
Department of Chemistry, Division of Molecular Imaging and Photonics, KU Leuven, Celestijnenlaan 200F, B-3001 Leuven, Belgium
E-mail: johannes.seibel@kuleuven.be, steven.defeyter@kuleuven.be
S. Eyley and W. Thielemans
Department of Chemical Engineering, Sustainable Materials Lab, KU Leuven, campus Kulak Kortrijk, E. Sabbelaan 53, 8500 Kortrijk, Belgium

Contents:

1. Comparison of this work with the reported strategies for the CFG (Table S1).
2. Band position shift in graphene exposed to the different candidate solvents (Figure S1).
3. Band position shift in the DMSO-doped graphene as a function of DMSO exposure time (Figure S2).
4. Stability and de-doping of DMSO-doped graphene (Figure S3).
5. Raman maps of the pristine and functionalized SLG/SiO_2 using different solvents for the NBD grafting inks (Figure S4).
6. Concentration-dependent functionalization of SLG/SiO_2 by the NBD-DMSO grafting ink (Figure S5).
7. I(D)/I(G) ratio maps for various samples of SLG/SiO_2 exposed to NBD-DMSO grafting ink for 10 min (Figure S6).
8. Raman band shift as a function of exposure time of SLG/SiO_2 to the grafting ink (Figure S7).
9. AFM characterization of the grafted layers for functionalized graphene (Figure S8).

10. STM characterization of the grafting layers for functionalized graphene (Figure S9).
11. Representative Raman spectra of the sample functionalized by direct writing (Figure S10).
12. Raman measurement of HOPG treated with the grafting ink (Figure S11).
13. SEM images of functionalized SLG/SiO_2 (Figure S12).
14. EDS mapping of functionalized SLG/SiO_2 (Figure S13).
15. XPS spectra of NBD grafted SLG and the restored SLG by annealing (Figure S14).
16. XPS spectra of BBD and DCBD grafted SLG (Figure S15)
17. Stability of the grafting ink for effective CFG (Figure S16).
18. The time-dependent NMR spectra of NBD in DMSO-d_6 at RT (Figure S17).
19. High-resolution ^{1}H-NMR spectrum of NBD after 14 days in DMSO-d_6 (Figure S18).
20. MS-spectrum of NBD after 14 days in DMSO-d_6 (Figure S19).
21. Chemical structures of the byproducts generated in the degradation of NBD (Scheme S1).
22. Composition of the NBD solution over time (Table S2).
23. Reversible functionalization of SLG/SiO_2 by the grafting ink (80 mM NBD) (Figure S20).
24. References.

Table S1. Comparison of this work with the reported strategies for the CFG, using SLG/SiO_2.

Strategies for CFG	I(D)/I(G)	Reaction time	Key factor	T/℃	Environment
**Solvation activating (*this work*)**	Beyond 5 (Failure of T-K relation)	60 s ~ 1 h	Solvent	RT	Ambient
**Electrochemical** [1,2]	4.0 ~ 4.5	30 s ~ overnight	Electric field	RT	Ambient
**Alkali metal doping** [3,4]	2.8	~ 1.5 h	Alkali metal	RT	Glovebox with Ar (< 0.1 ppm O_2, < 0.1 ppm H_2O)
**Photoactivation** [5]	0.6	120 s	Laser	RT	Ambient
**SDS[a)]-mediation** [6–8]	0.3 ~ 1.4	7 h ~ 16 h	SDS	30 ~ 45	Ambient
**Extra reducing reagent** [9,10]	~ 3	10 min	Reducing reagent	RT	Ambient

a) SDS = sodium dodecyl sulphate

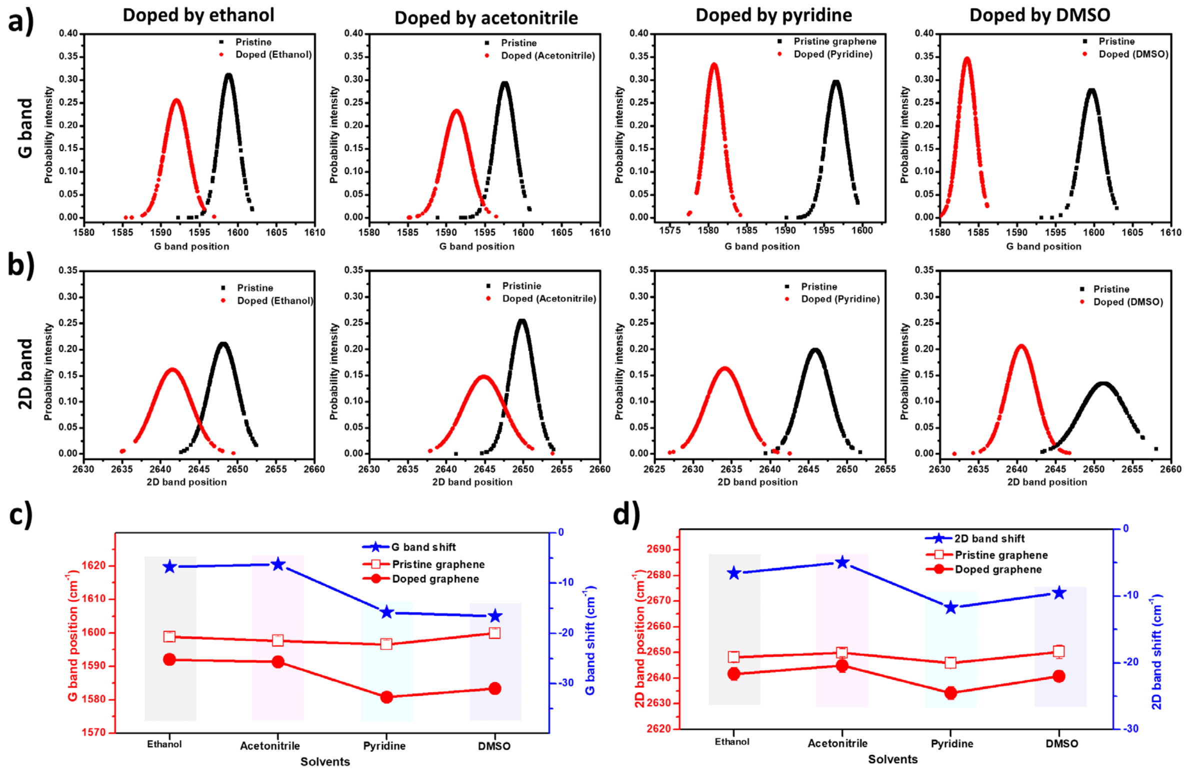

Figure S1. Band positions shift in graphene exposed to the different candidate solvents, as obtained by Raman mapping (400 points (20×20), 50 μm×50 μm). The pristine SLG/SiO_2 samples were first characterized by Raman spectroscopy. The solvent exposure time was fixed to 10 min. Histogram of (a) G band position and (b) 2D band position of pristine (black) and exposed (red) graphene. (c) G band position and shift, and (d) 2D band position and shift, for the different solvents.

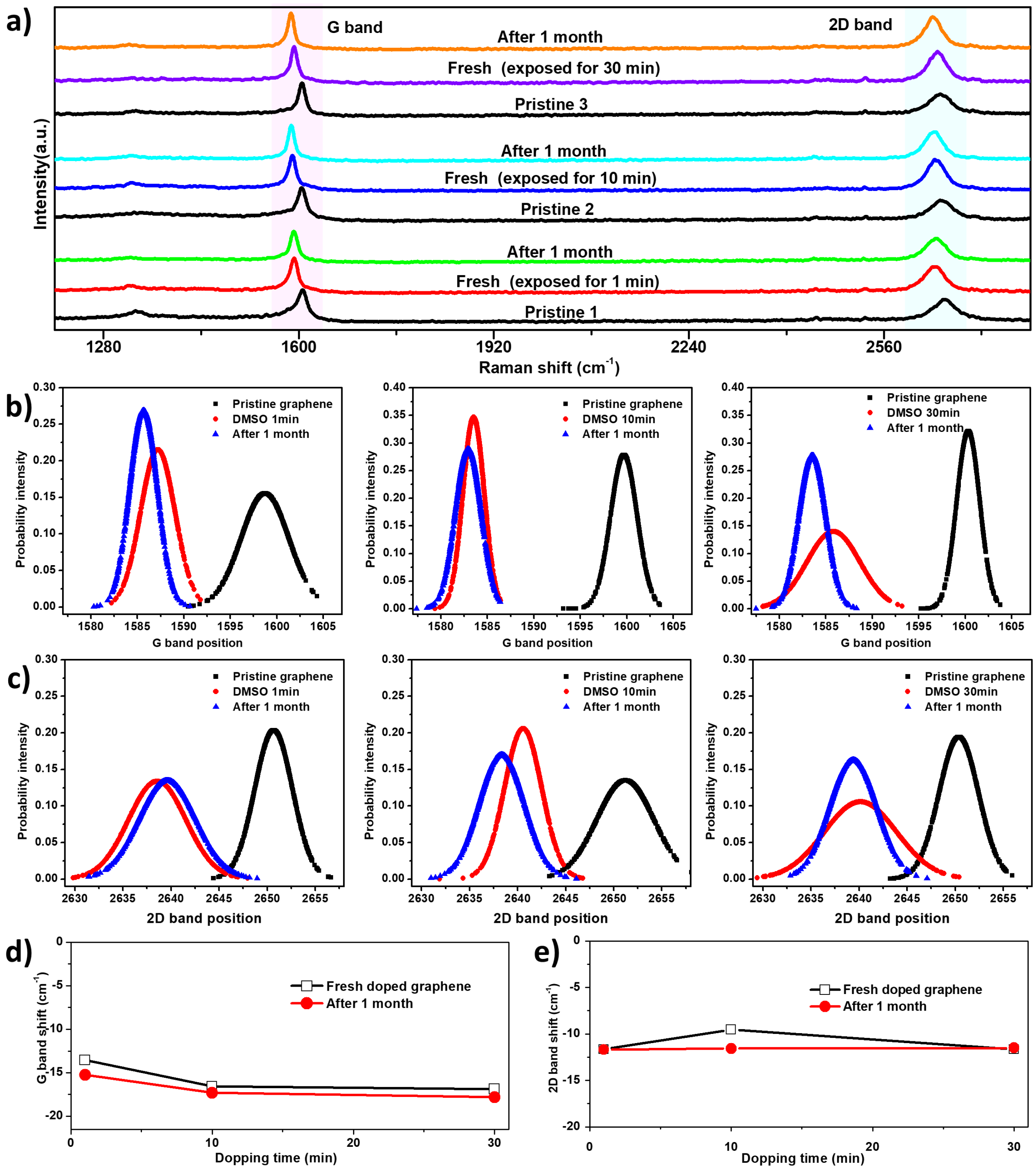

Figure S2. Band position shift in DMSO-doped graphene as a function of DMSO exposure time. Raman measurements were performed for the samples before exposure to DMSO (pristine SLG/SiO_2), after exposure for a fixed time (1, 10 and 30 min), and the exposed graphene stored at RT for one month in nitrogen atmosphere. (a) Representative Raman spectra. Histogram of (b) G band position and (c) 2D band position of pristine graphene (black), graphene exposed to DMSO for 1, 10 and 30 min (red), and the exposed graphene stored for one month in nitrogen atmosphere (blue). (d) G band shift, and (e) 2D band shift for graphene exposed to DMSO for 1, 10 and 30 min, immediately after exposure (black) and after storage for one month (red).

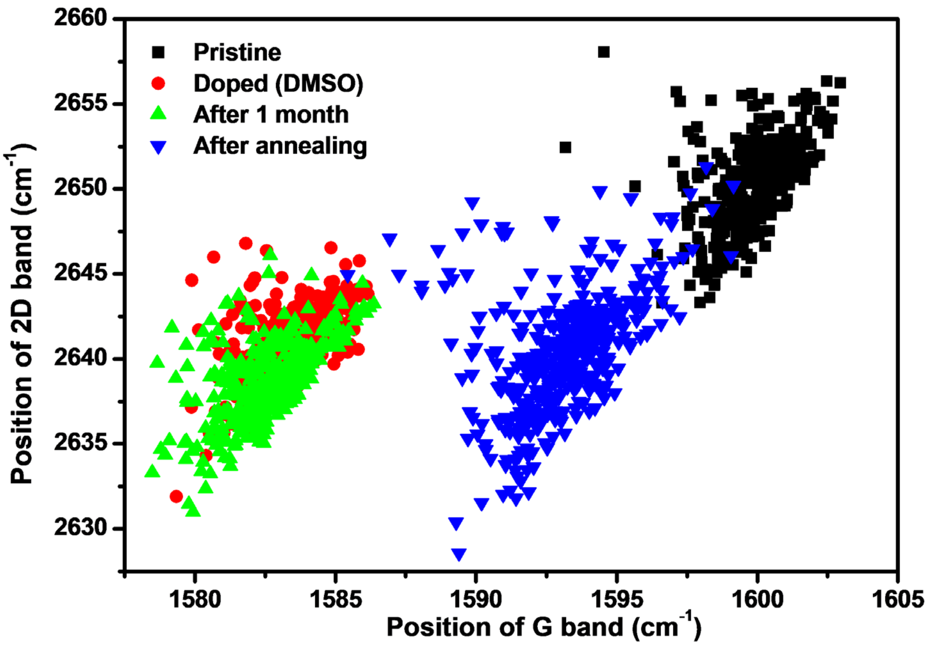

Figure S3. Stability and de-doping of DMSO-doped graphene. The scatter plot of the Raman 2D band versus G band positions for pristine graphene (SLG/SiO_2) (black), DMSO-doped graphene (exposure time 10 min) (red), the doped graphene stored in nitrogen atmosphere for 1 month (green) and DMSO-doped graphene annealed at 400 °C (blue), leading to de-doping. Note that a small shift of the G and 2D bands remains for thermally treated samples after annealing, owing to the impact of the treatment on the state of graphene lattice.[11,12]

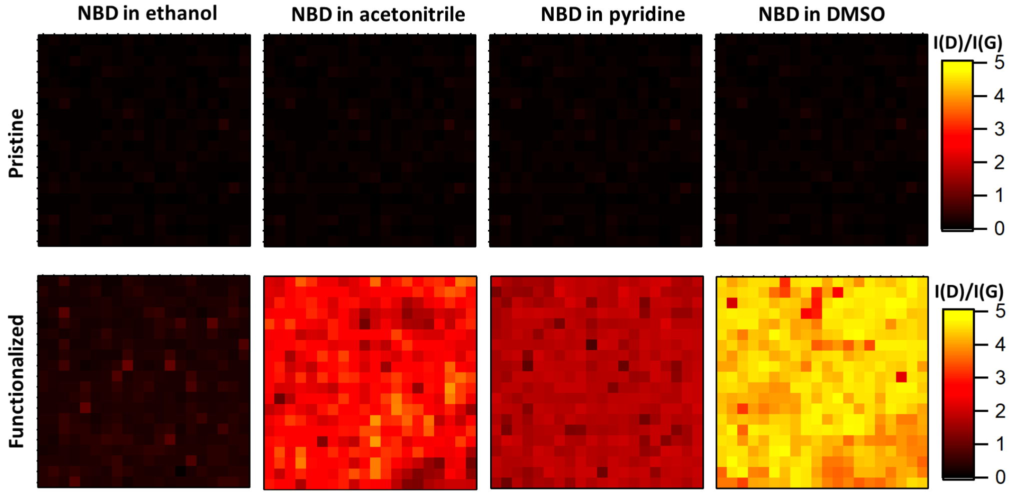

Figure S4. Raman maps of the pristine and functionalized SLG/SiO_2 using different solvents for the NBD grafting inks. The concentration of NBD in acetonitrile, pyridine and DMSO is 40 mM. A saturated solution was prepared in ethanol (<10 mM). The functionalization time was fixed at 10 min. The Raman maps reflect the I(D)/I(G) ratios.

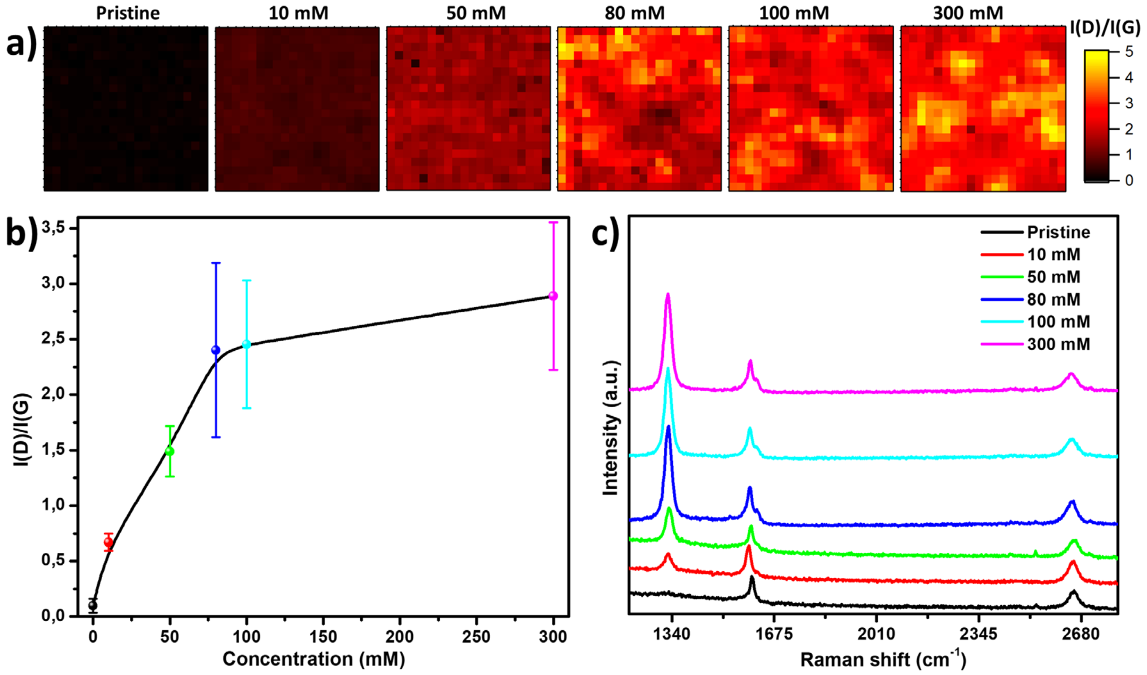

Figure S5. Concentration-dependent functionalization of SLG/SiO_2 by the NBD-DMSO grafting ink. The NBD concentration ranges from 10 mM to 300 mM. The functionalization time was fixed at 1 min. (a) Raman maps of pristine graphene and NBD-DMSO exposed graphene for various concentrations of NBD. The Raman maps reflect the I(D)/I(G) ratio. (b) Average value of I(D)/I(G) as a function of NBD concentration. (c) Representative Raman spectra of the concentration-dependent functionalized SLG/SiO_2 samples.

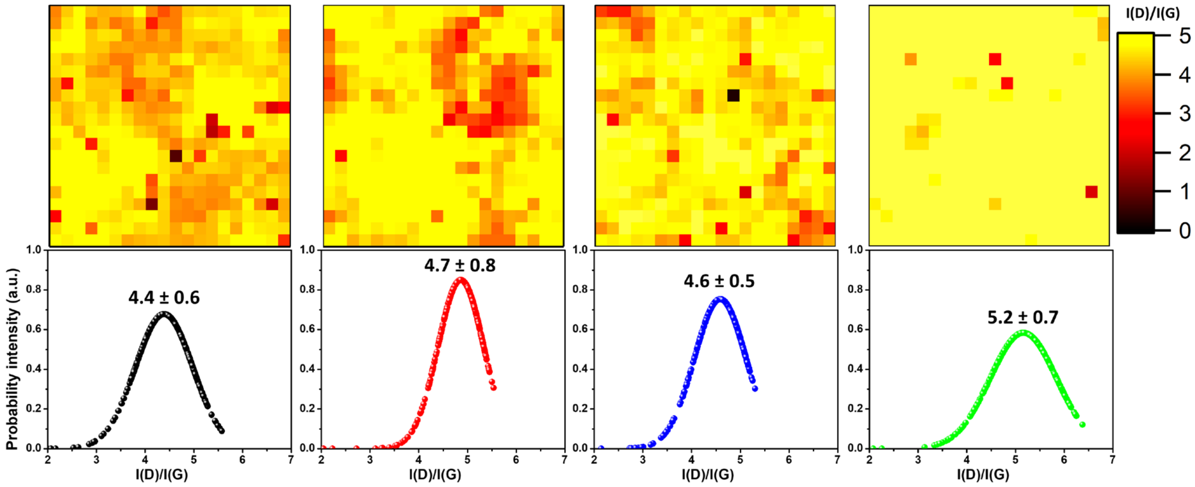

Figure S6. I(D)/I(G) ratio maps for various samples of SLG/SiO_2 exposed to NBD-DMSO for 10 min, reflecting the sample to sample variation. The concentration of NBD is 80 mM.

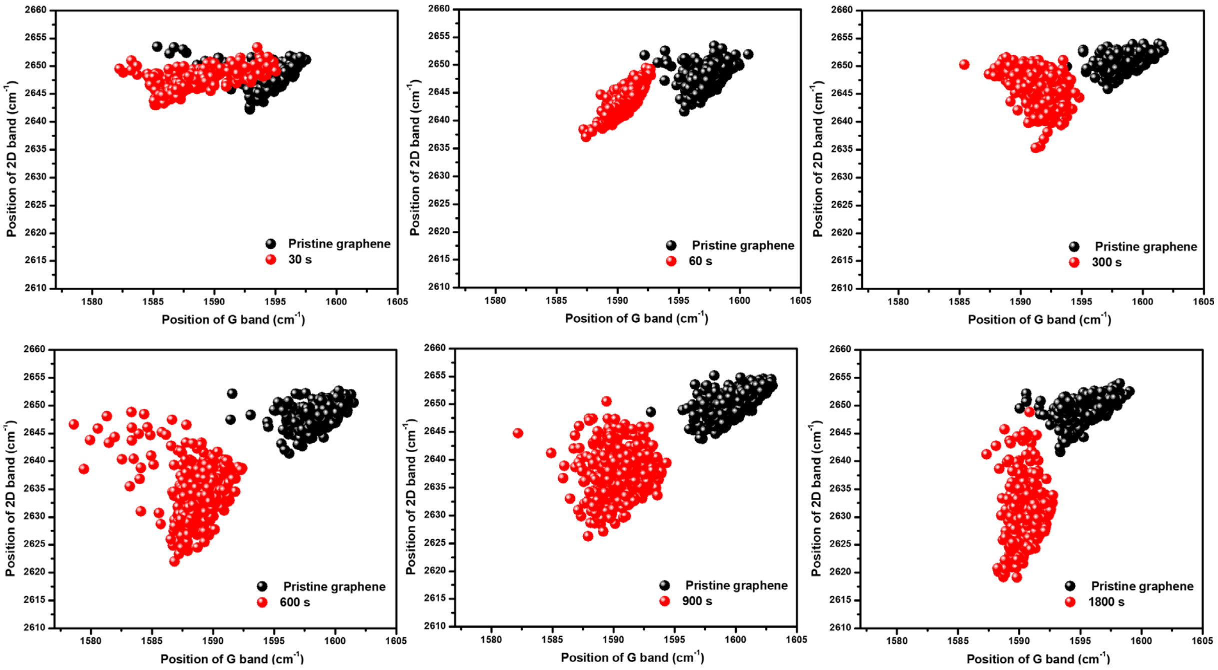

Figure S7. Raman band shift as a function of exposure time of SLG/SiO_2 to the grafting ink (80 mM NBD in DMSO). Scatter plots of 2D band versus G band positions for pristine SLG/SiO_2 and SLG/SiO_2 exposed to the grafting ink for 30 s, 60 s, 300 s, 600 s, 900 s, or 1800 s.

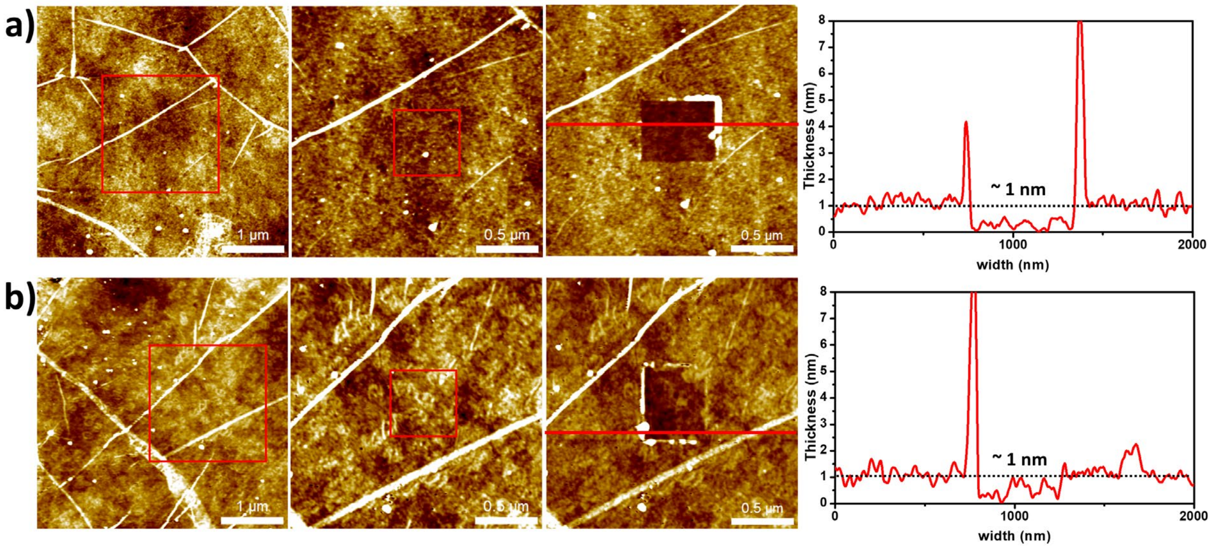

Figure S8. AFM characterization of the grafted layers for the functionalized graphene. The functionalized SLG/SiO_2 samples exposured to NBD-DMSO grafting ink (80 mM NBD) for (a) 10 min and (b) 30 min. A zoomed-in area (0.5 µm by 0.5 µm) was removed by AFM-nanolithography to reveal the layer thickness, shown by the line profile on the right. Under both conditions, the layer thicknesses is about ~1.0 nm.

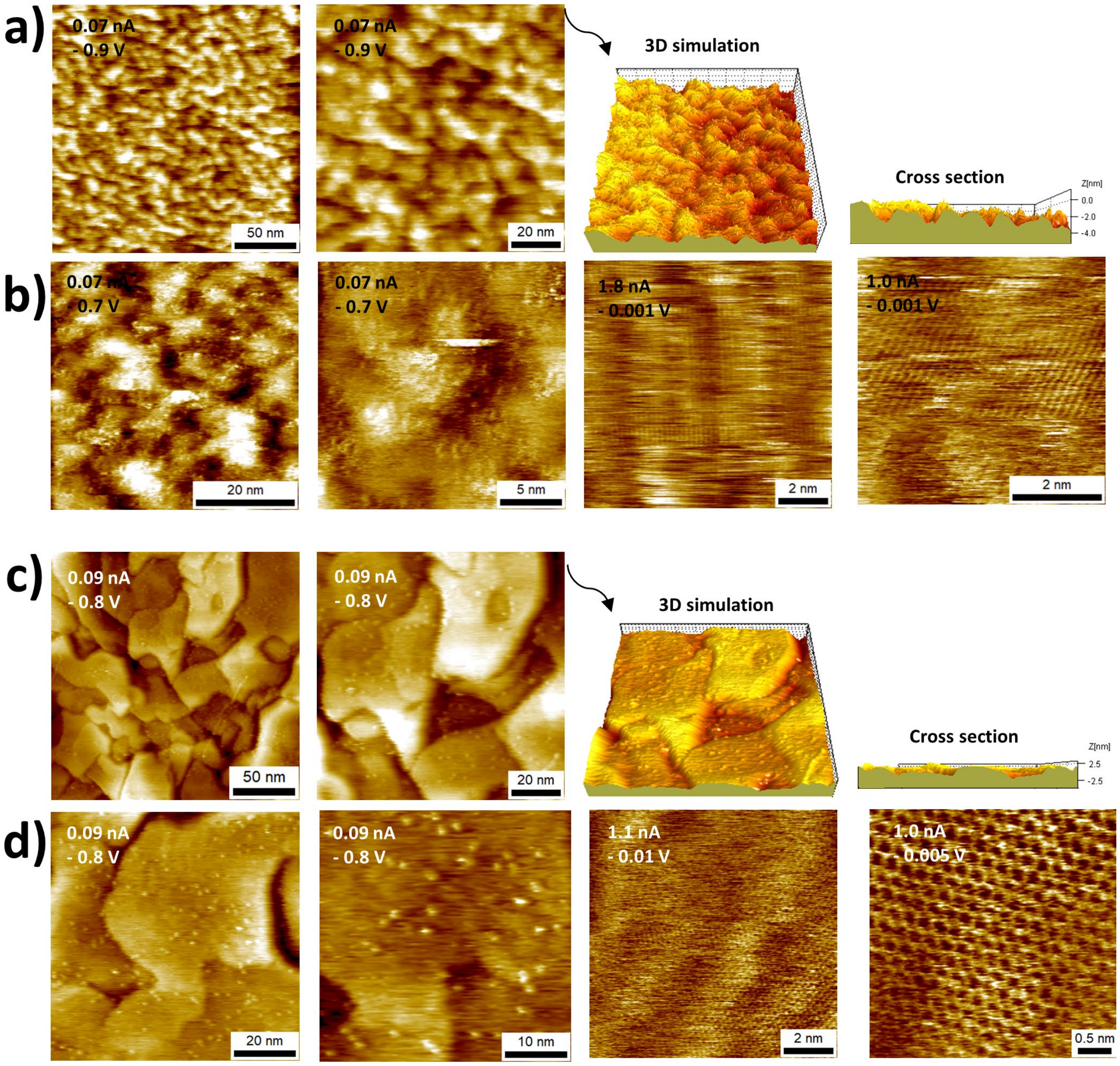

Figure S9. STM characterization of the grafted layers for the functionalized graphene. STM images of (a, b) functionalized SLG/SiO_2 and (c, d) functionalized SLG/Cu using NBD-DMSO grafting ink (80mM NBD). Exposure time is 10 min. The STM scanning parameters are indicated on the top-left corner of the STM images. A high tunneling current and low voltage is used to deliberately remove the grafting molecules, revealing the graphene lattice. Functionalized SLG/Cu leads to better resolution STM images than functionalized SLG/SiO_2 because of the better electrical conductivity of the former samples. Note that the apparent roughness of SLG is due to the roughness of the underlying substrate.[13–16]

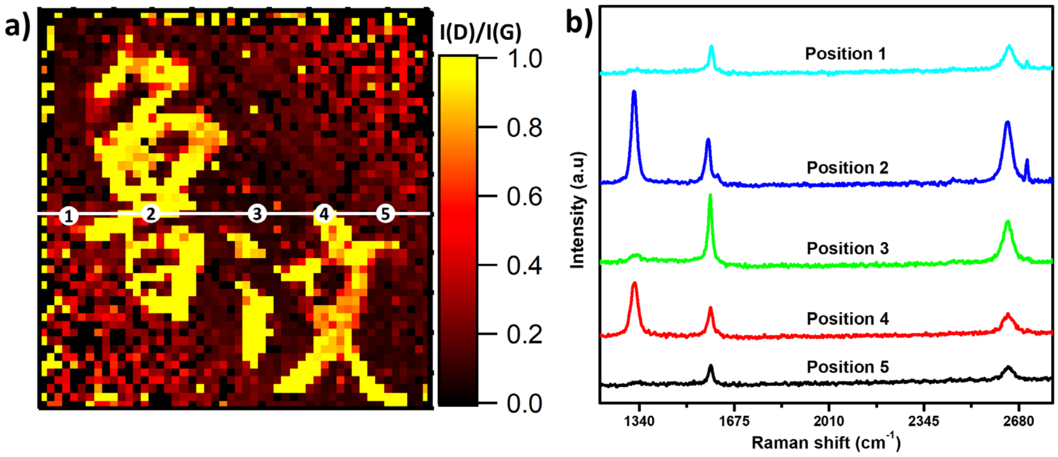

Figure S10. Representative Raman spectra of the sample functionalized by direct writing. (a) Raman map of the I(D)/I(G) ratios showing the Chinese calligraphy. A Chinese brush was dipped in the grafting ink (80 mM NBD) to write on the graphene sample (1 by 1 cm^2 SLG/SiO_2). After drying the sample in ambient conditions, Raman mapping (2500 points (50×50), 1 by 1 cm^2) revealed the Chinese name for Leuven. (b) Representative Raman spectra at different positions in the sample functionalized by direct writing.

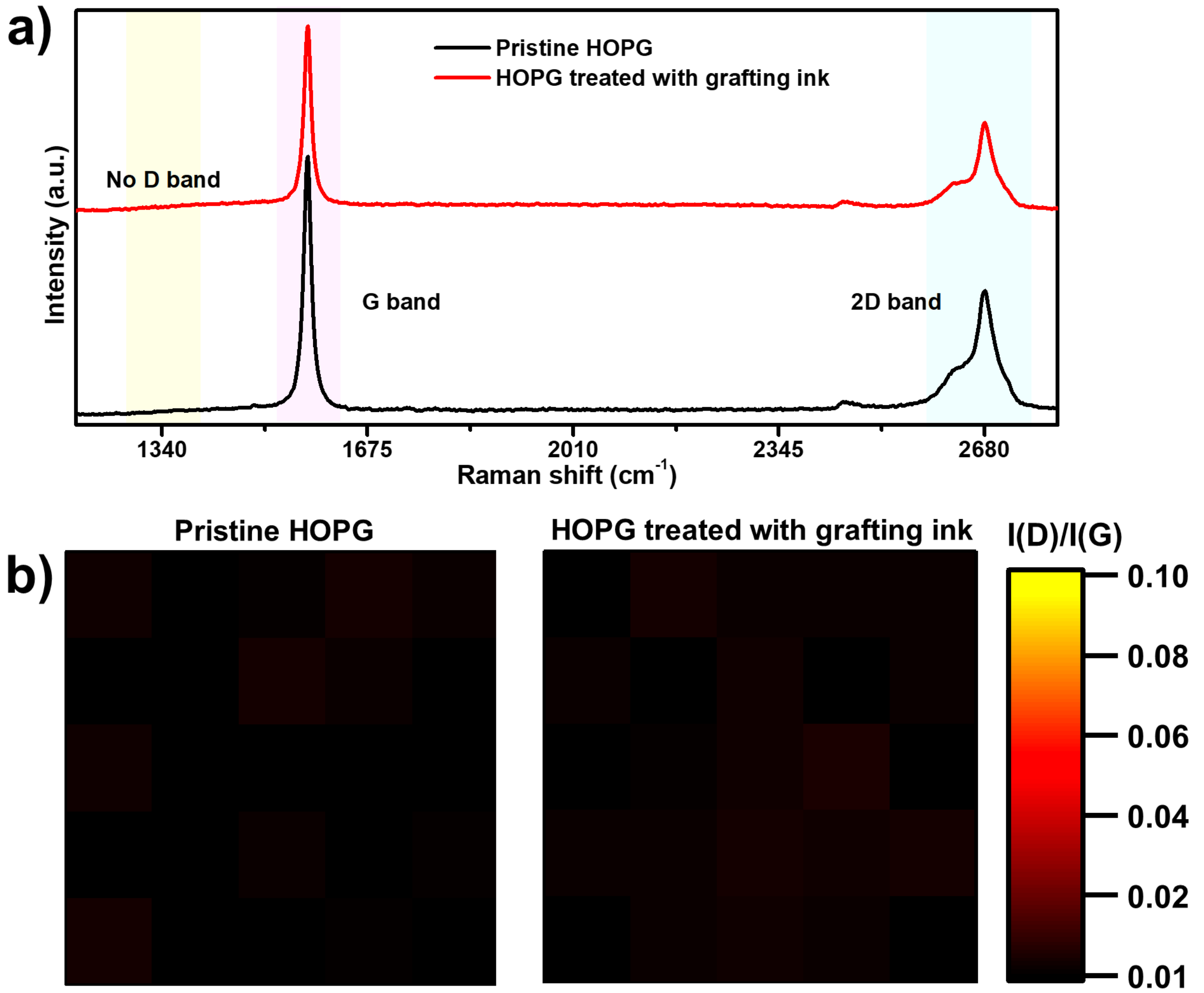

Figure S11. Raman measurement of HOPG treated with the grafting ink (80 mM NBD in DMSO) for 10 min. (a) Representative Raman spectra of treated and pristine HOPG. (b) Raman maps of the I(D)/I(G) ratio. Raman mapping condition: 25 points (5 by 5), 50 µm by 50 µm.

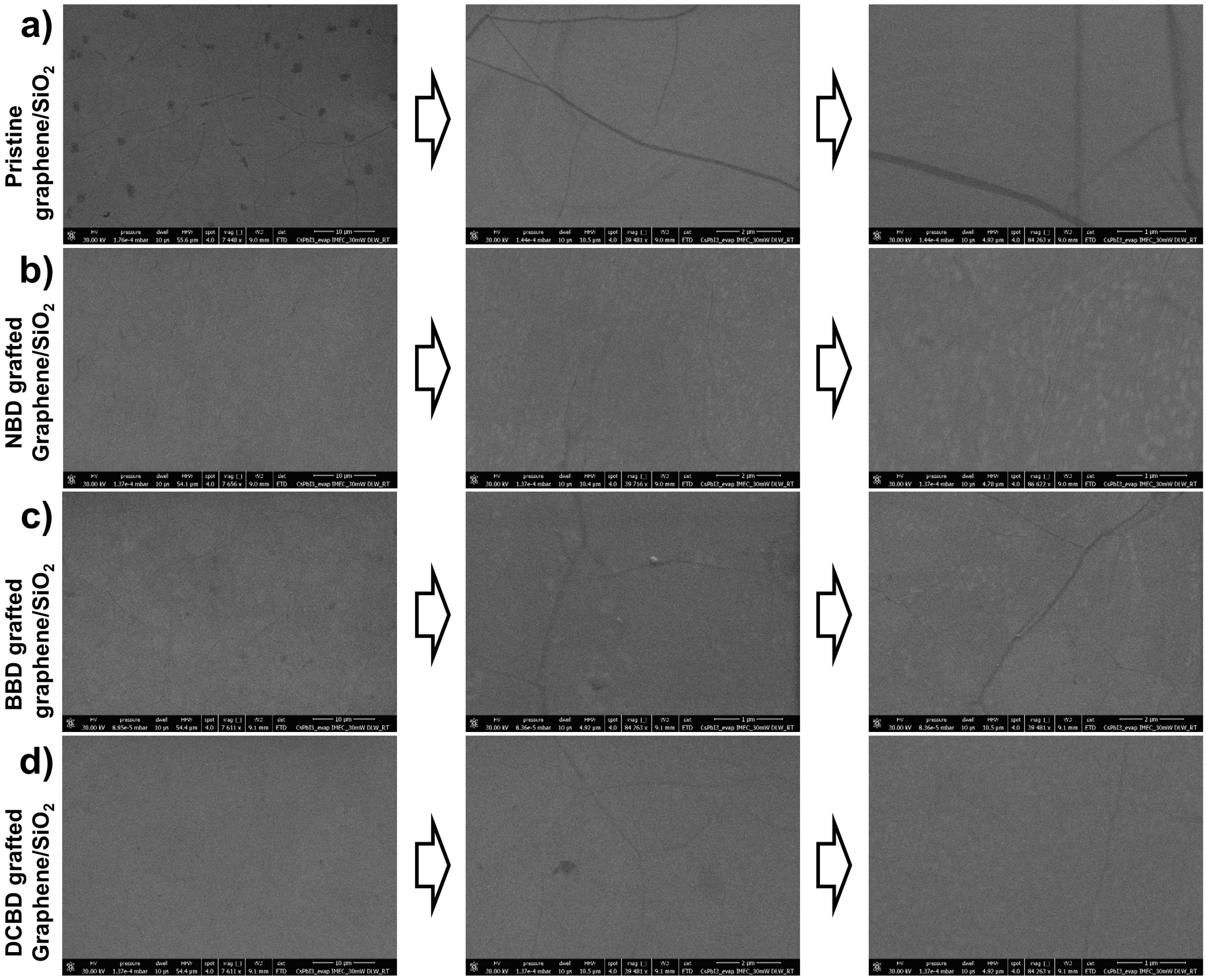

Figure S12. SEM images of functionalized SLG/SiO_2. (a) Pristine SLG/SiO_2. (b) NBD grafted SLG/SiO_2. (c) BBD grafted SLG/SiO_2. (d) DCBD grafted SLG/SiO_2. Functionalization conditions for preparing the grafted SLG: grafting ink (80 mM NBD) /functionalization time (10 min); grafting ink (80 mM BBD) / functionalization time (30 min); grafting ink (80 mM DCBD) / functionalization time (60 min). The SEM images were recorded with operating voltage 30 kV. Scale bar: 10 μm (left column), 2 μm (middle column) and 1 μm (right column).

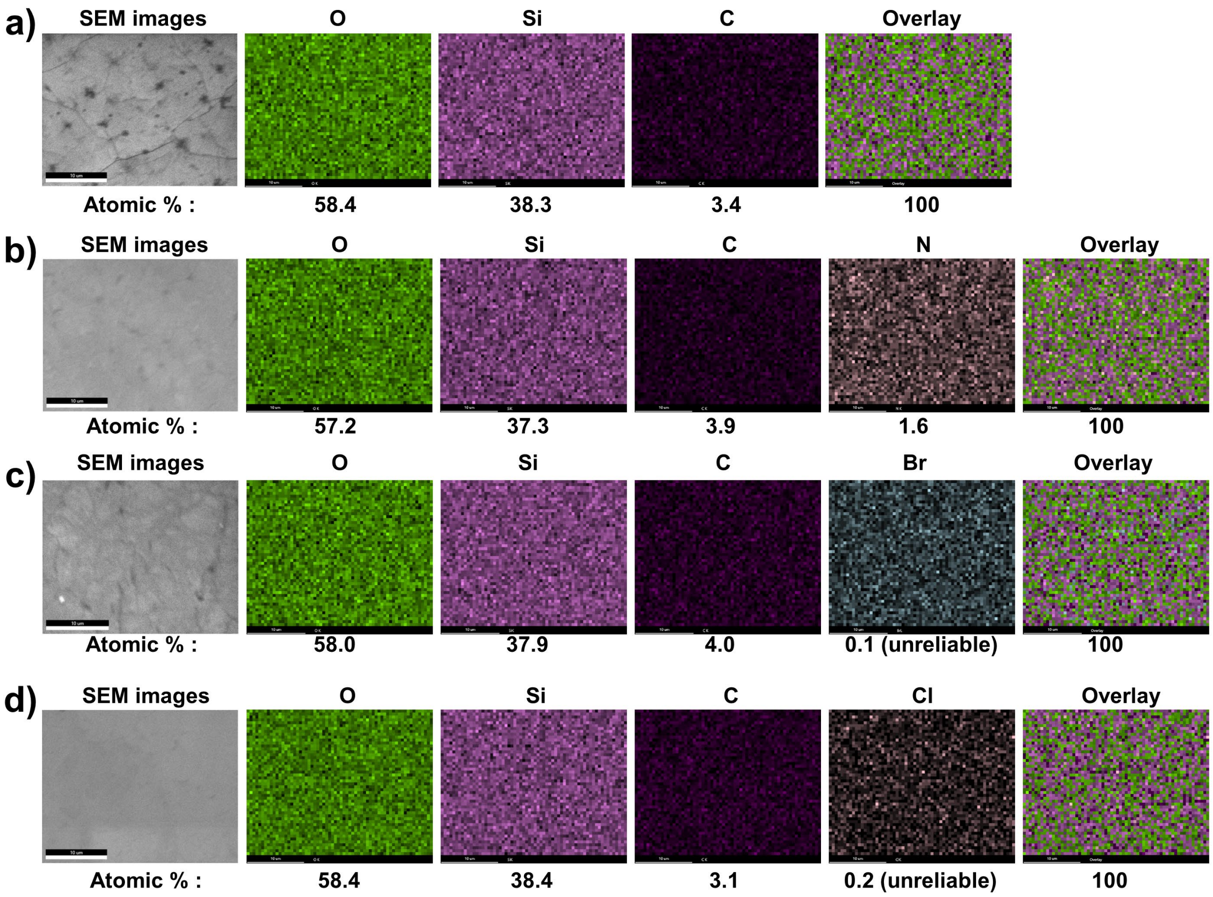

Figure S13. EDS mapping of functionalized SLG/SiO_2. (a) EDS element distributions of oxygen (O), silicon (Si) and carbon (C) for the pristine SLG/SiO_2. (b) EDS element distributions of O, Si, C and nitrogen (N) for NBD functionalized SLG/SiO_2. (c) EDS element distributions of O, Si, C and bromine (Br) for BBD functionalized SLG/SiO_2. (d) EDS element distributions of O, Si, C and chlorine (Cl) for DCBD functionalized SLG/SiO_2. Functionalization conditions for preparing the grafted SLG: grafting ink (80 mM NBD) /functionalization time (10 min); grafting ink (80 mM BBD) / functionalization time (30 min); grafting ink (80 mM DCBD) / functionalization time (60 min). EDS mapping was recorded with operating voltage of 5 kV. Scale bar: 10 μm. Note that the element signals are dominated by the underlying substrate (O and Si from SiO_2/Si wafer), because of the significant penetration depth of the incident electron beam compared with the thickness of the organic layer, in combination with the limited sensitivity of EDS. Br and Cl intensity do not exceed background levels. To obtain additional information on the chemical composition of the grafting layers, XPS measurements were performed (See Figure S14 and S15).

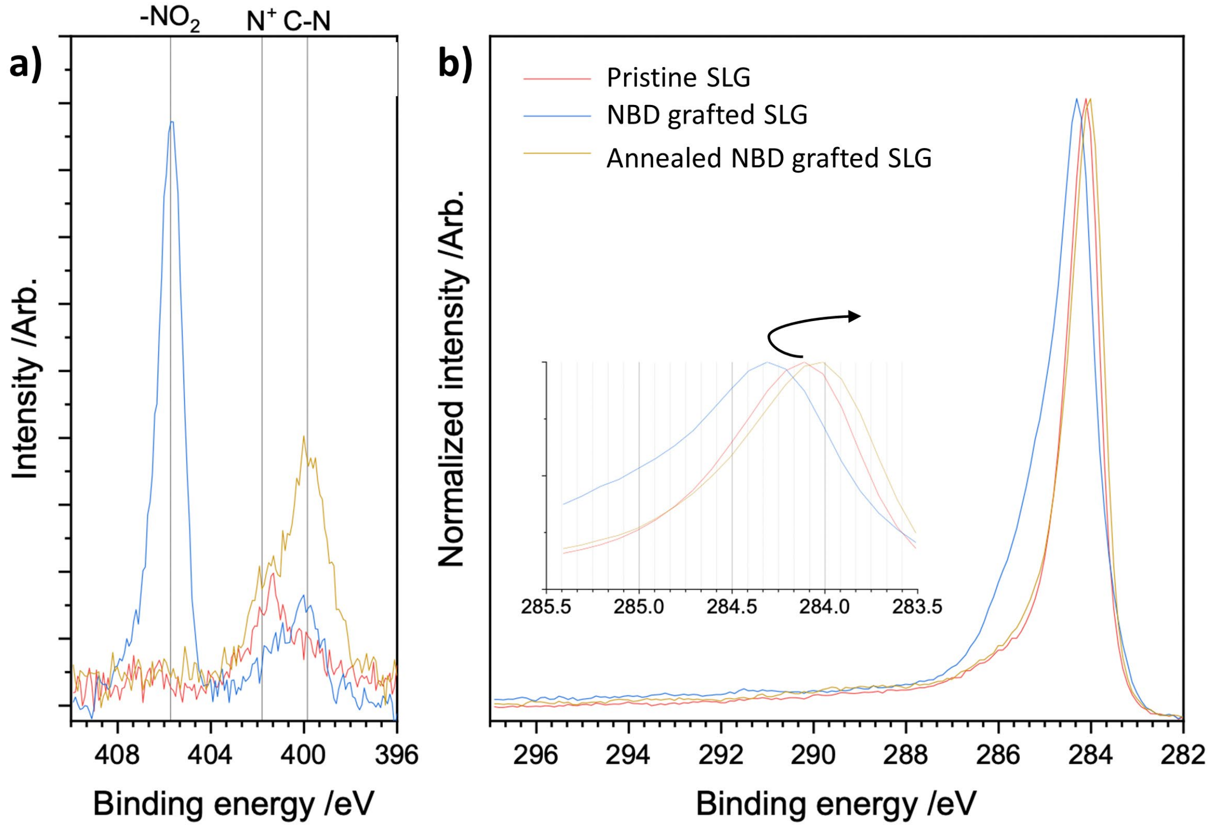

Figure S14. XPS spectra of the NBD grafted SLG and the restored SLG after annealing to 400 °C in a nitrogen atmosphere. (a) Nitrogen 1s and (b) carbon 1s spectra of pristine SLG, NBD grafted SLG, and the latter heated to 400 °C in a nitrogen atmosphere. The NBD grafted SLG is prepared by the functionalization of SLG/SiO_2 using the grafting ink (80 mM NBD in DMSO) for 10 min. Annealing NBD grafted SLG at 400 °C in a nitrogen atmosphere regenerates pristine SLG.

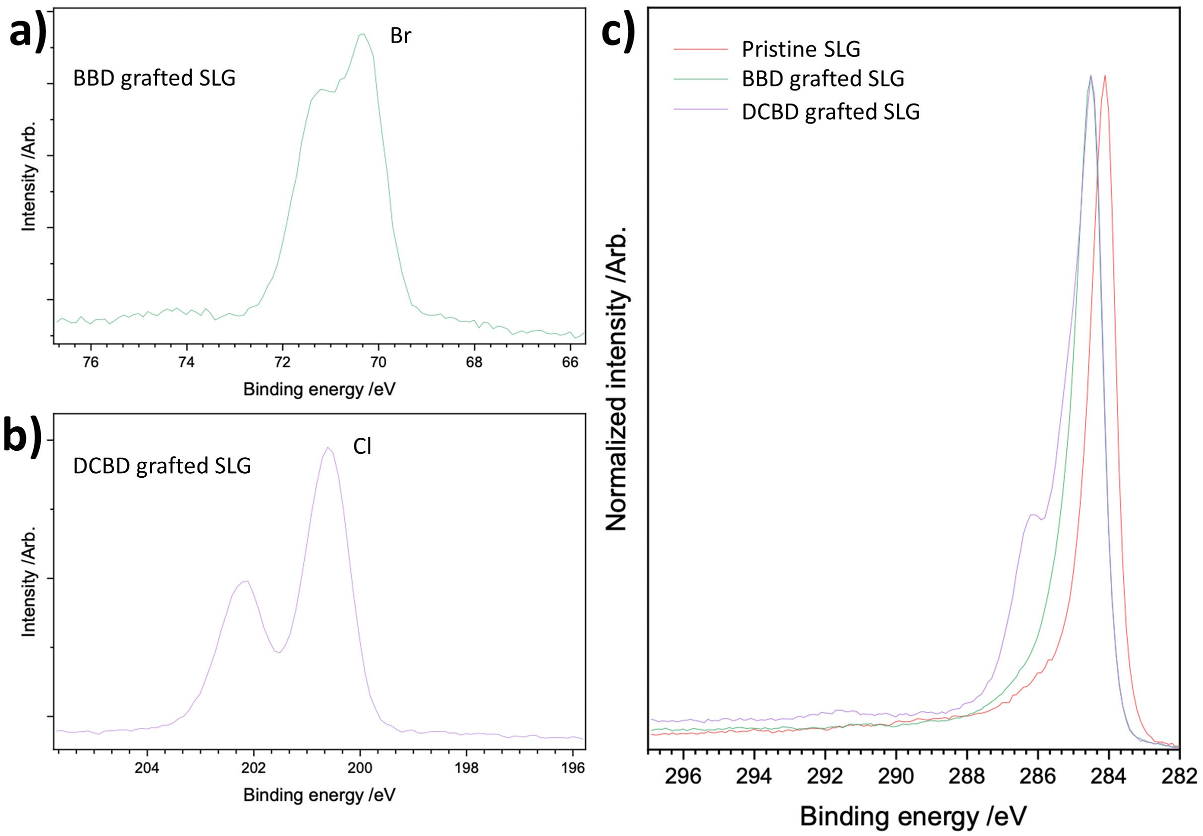

Figure S15. XPS spectra of the BBD and DCBD grafted SLG. (a) Bromine 3d, (b) chlorine 2p and (c) carbon 1s spectra of the pristine, BBD grafted and DCBD grafted SLG. The BBD grafted SLG is prepared by the functionalization of SLG/SiO_2 using the grafting ink (80 mM NBD in DMSO) for 30 min. The DCBD grafted SLG is prepared by the functionalization of SLG/SiO_2 using the grafting ink (80 mM NBD in DMSO) for 60 min.

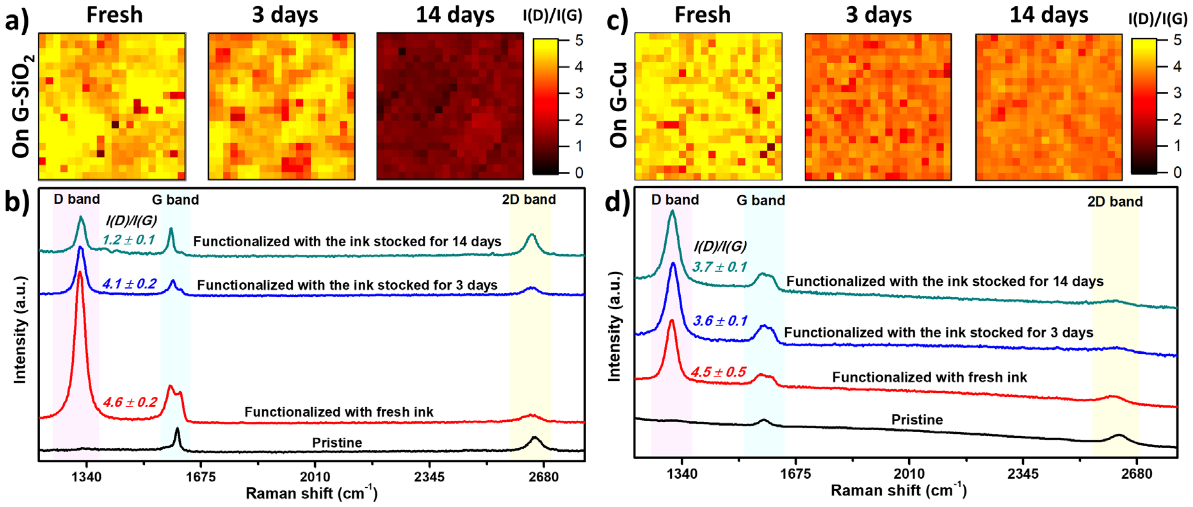

Figure S16. Stability of the grafting ink for effective CFG. (a) Raman maps and (b) corresponding representative Raman spectra of SLG/SiO_2 functionalized using fresh NBD-DMSO grafting ink (80mM NBD), and the same grafting ink aged for 3 days and 14 days, respectively. (c) Raman maps and (d) corresponding representative Raman spectra of the SLG/Cu functionalized using fresh NBD-DMSO grafting ink (80mM NBD), and the same grafting ink aged for 3 days and 14 days, respectively. Functionalization time 10 min.

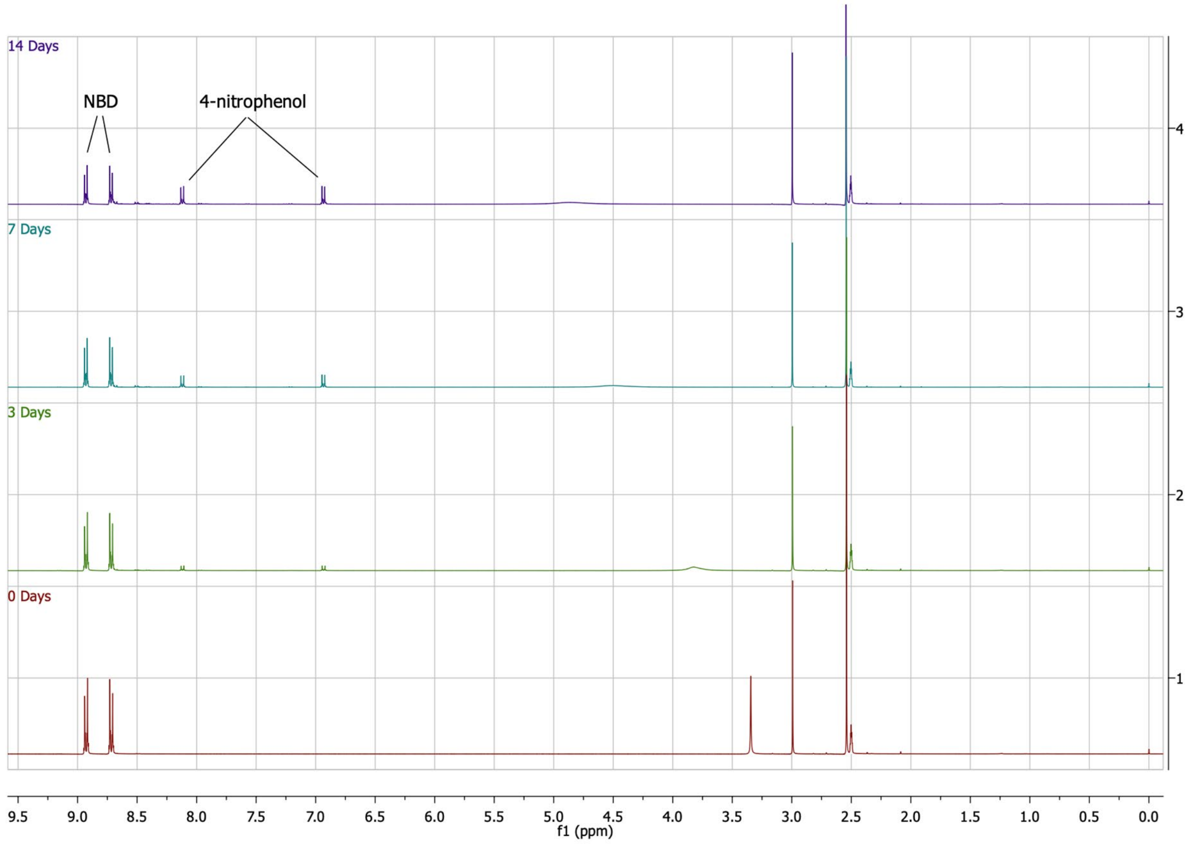

Figure S17. The time-dependent ^{1}H-NMR spectra of NBD in DMSO-d_6 at RT. 80 mM NBD solution (in DMSO-d_6) was stored at ambient condition. Degradation of NBD was followed over time with ^{1}H-NMR in DMSO-d_6: fresh solution (red), after 3 days (green), after 7 days (light blue), after 14 days (dark blue). A gradual decrease of the (δ8.93, 2H; 8.72, 2H) is observed. 4-Nitrophenol (δ8.12, 2H; δ6.94, 2H) is formed as main byproduct,[17] consistent with previous reports.[18,19] It was reported that the origin of the phenolic oxygen atom of 4-nitrophenol may be from the DMSO, air and/or traces of water.[18]

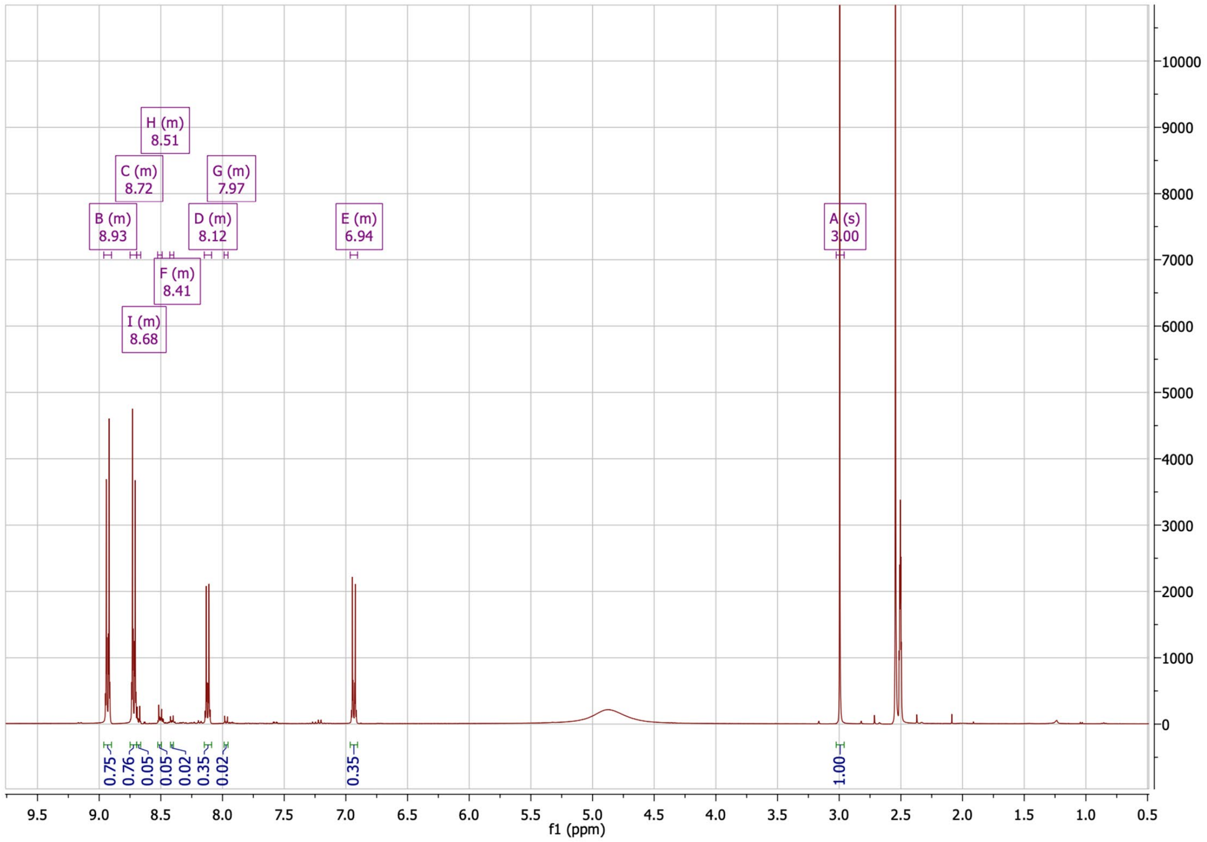

Figure S18. High resolution ^{1}H-NMR spectrum of a 80 mM NBD solution in DMSO-d_6 stored for 14 days at room temperature. 80 mM NBD solution (in DMSO-d_6) stored at ambient condition for 14 days. In addition to 4-nitrophenol (δ8.12, 2H; δ6.94, 2H), the main degradation product, other degradation products are 1-(methylsulfinyl)-4-nitrobenzene (δ8.41, 2H; δ7.97, 2H) and 1-(methylsulfonyl)-4-nitrobenzene (δ8.68, 2H; δ8.51, 2H).

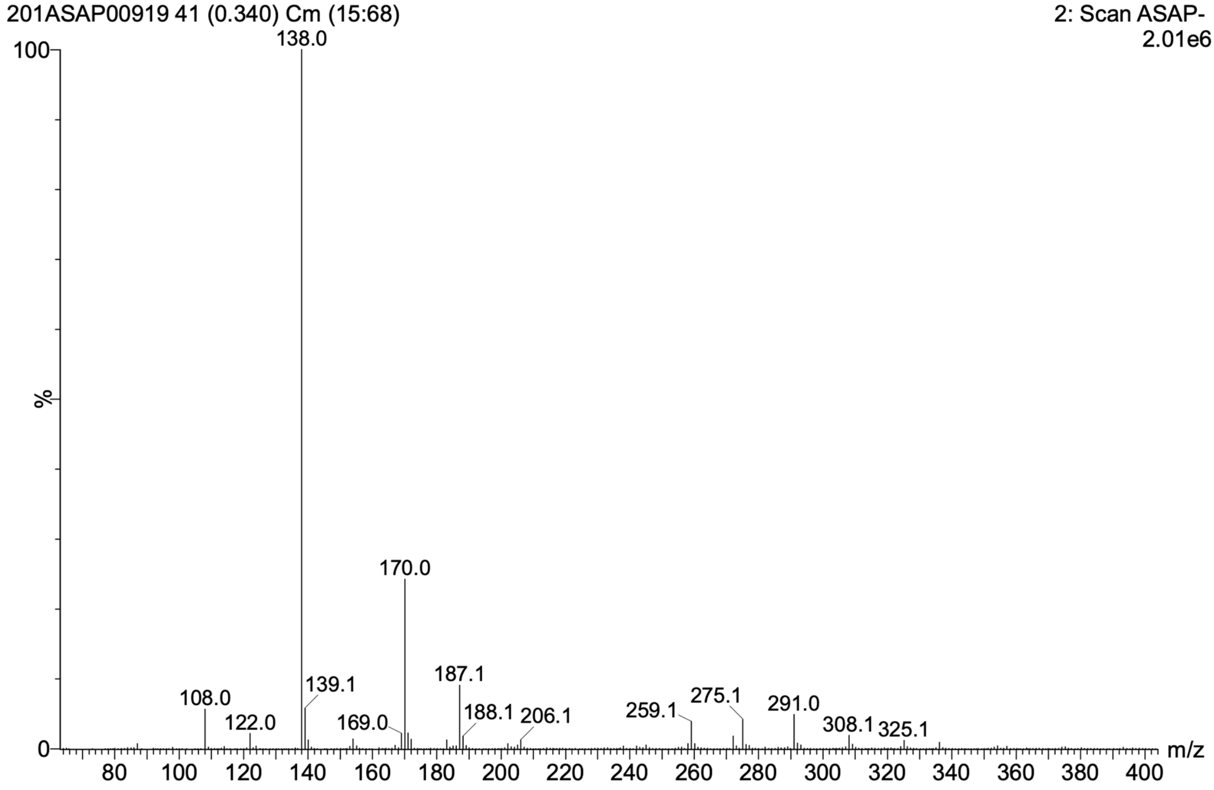

Figure S19. MS-spectrum of a 80 mM NBD solution in DMSO-d_6 stored for 14 days at room temperature. As NBD degrades upon injection, the main peaks in the MS-spectrum originate from 4-nitrophenol (m/z=138.0, P-H^+; m/z=108.0, P-H^+-NO). The peak at m/z=170.0 originates from 1-(methylsulfinyl)-4-nitrobenzene (m/z=170.0, P-CH_3^+; m/z=275.1, P+BF_4^-) and 4-nitrothioanisole (m/z=170.0, P-D^+; m/z=259.1, P+BF_4^-). 1-(methylsulfonyl)-4-nitrobenzene (m/z=291.0, P+BF_4^-) is also observed. All the byproducts observed by ^{1}H-NMR (**Figure S17** and **Figure S18**) are confirmed by MS.

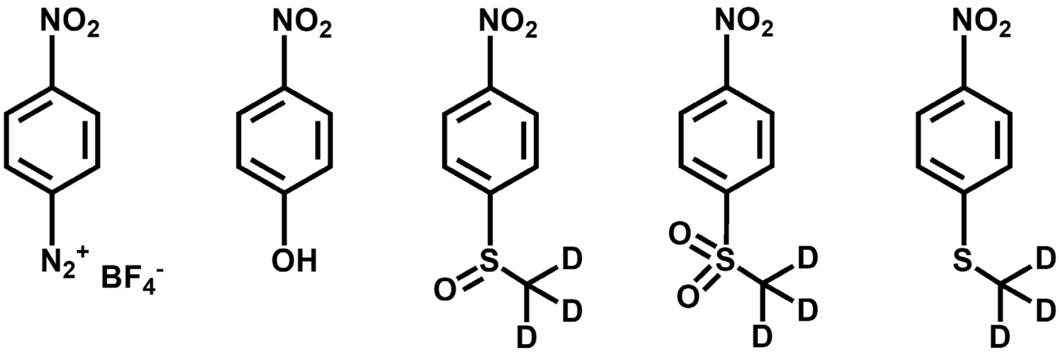

NBD 1 2 3 4

Scheme S1. Chemical structures of the byproducts generated in the degradation of NBD in *DMSO-d_6*. 4-nitrophenol (**1**), 1-(methylsulfinyl)-4-nitrobenzene (**2**), 1-(methylsulfonyl)-4-nitrobenzene (**3**) and 4-nitrothioanisole (**4**). These byproducts were determined by NMR and MS measurements (**Figure S17**, **Figure S18** and **Figure S19**).

Table S2. Composition of the NBD solution over time. The percentages are calculated from the amount of NBD that was added at the start of the experiment using dimethylsulfone as standard. The quantification results are based on the NMR results (**Figure S17** and **Figure S18**)

Stored time of 80 mM NBD in DMSO (at RT)	Percentage of NBD (%)	Percentage of the byproducts		
		4-nitrophenol	1-(methylsulfinyl)-4-nitrobenzene	1-(Methylsulfonyl)-4-nitrobenzene
Fresh	100%	0%	0%	0%
3 days	90%	7%	1%	2%
7 days	76%	18%	2%	4%
14 days	59%	28%	2%	4%

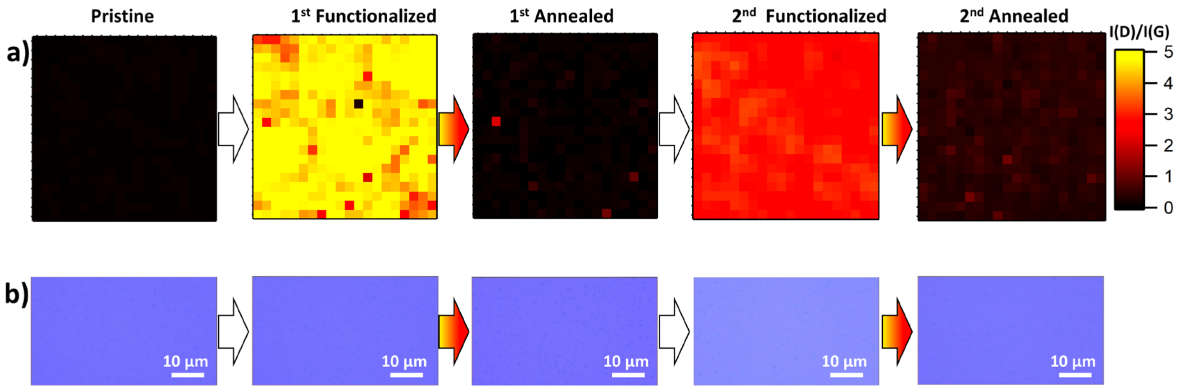

Figure S20. Reversible functionalization of SLG/SiO_2 by the grafting ink (80 mM NBD in DMSO). Functionalization time fixed at 10min. Pristine SLG/SiO_2 was recovered by annealing at 400 °C. (a) Raman maps of the I(D)/I(G) ratios of two functionalization/de-functionalization cycles. (b) Corresponding optical images indicate that the reversible CFG preserves the quality of graphene on SiO_2. The blue contrast is typical for SLG/SiO_2.

References:

[1] C. J. Shih, Q. H. Wang, Z. Jin, G. L. C. Paulus, D. Blankschtein, P. Jarillo-Herrero, M. S. Strano, *Nano Lett.* **2013**, *13*, 809.

[2] Z. Xia, F. Leonardi, M. Gobbi, Y. Liu, V. Bellani, A. Liscio, A. Kovtun, R. Li, X. Feng, E. Orgiu, P. Samorì, E. Treossi, V. Palermo, *ACS Nano* **2016**, *10*, 7125.

[3] T. Wei, M. Kohring, M. Chen, S. Yang, H. B. Weber, F. Hauke, A. Hirsch, *Angew. Chemie* **2020**, *132*, 5651.

[4] T. Wei, M. Kohring, H. B. Weber, F. Hauke, A. Hirsch, *Nat. Commun.* **2021**, *12*, 1.

[5] Y. Li, W. Li, M. Wojcik, B. Wang, L. C. Lin, M. B. Raschke, K. Xu, *J. Phys. Chem. Lett.* **2019**, *10*, 4788.

[6] R. Sharma, J. H. Baik, C. J. Perera, M. S. Strano, *Nano Lett.* **2010**, *10*, 398.

[7] Z. Jin, T. P. McNicholas, C. J. Shih, Q. H. Wang, G. L. C. Paulus, A. J. Hilmer, S. Shimizu, M. S. Strano, *Chem. Mater.* **2011**, *23*, 3362.

[8] Q. H. Wang, Z. Jin, K. K. Kim, A. J. Hilmer, G. L. C. Paulus, C. J. Shih, M. H. Ham, J. D. Sanchez-Yamagishi, K. Watanabe, T. Taniguchi, J. Kong, P. Jarillo-Herrero, M. S. Strano, *Nat. Chem.* **2012**, *4*, 724.

[9] Y. Xia, C. Martin, J. Seibel, S. Eyley, W. Thielemans, M. Van Der Auweraer, K. S. Mali, S. De Feyter, *Nanoscale* **2020**, *12*, 11916.

[10] M. C. Rodríguez González, A. Leonhardt, H. Stadler, S. Eyley, W. Thielemans, S. De Gendt, K. S. Mali, S. De Feyter, *ACS Nano* **2021**, *15*, 10618.

[11] S. Ryu, L. Liu, S. Berciaud, Y. J. Yu, H. Liu, P. Kim, G. W. Flynn, L. E. Brus, *Nano Lett.* **2010**, *10*, 4944.

[12] S. D. Costa, J. E. Weis, O. Frank, M. Fridrichová, M. Kalbac, *RSC Adv.* **2016**, *6*, 72859.

[13] W. G. Cullen, M. Yamamoto, K. M. Burson, J. H. Chen, C. Jang, L. Li, M. S. Fuhrer, E. D. Williams, *Phys. Rev. Lett.* **2010**, *105*, 1.

[14] V. Geringer, M. Liebmann, T. Echtermeyer, S. Runte, M. Schmidt, R. Rückamp, M. C. Lemme, M. Morgenstern, *Phys. Rev. Lett.* **2009**, *102*, 1.

[15] E. Stolyarova, T. R. Kwang, S. Ryu, J. Maultzsch, P. Kim, L. E. Brus, T. F. Heinz, M. S. Hybertsen, G. W. Flynn, *Proc. Natl. Acad. Sci. U. S. A.* **2007**, *104*, 9209.

[16] M. Ishigami, J. H. Chen, W. G. Cullen, M. S. Fuhrer, E. D. Williams, *Nano Lett.* **2007**, *7*, 1643.

[17] ChemicalBook>CAS DataBase List>100-02-7More Spectrum> 4-Nitrophenol(100-02-7) 1H NMR, https://www.chemicalbook.com/SpectrumEN_100-02-7_1HNMR.htm.

[18] H. Yoji, G. H. Wahl, H. Zollinger, *Helv. Chim. Acta* **1976**, *59*, 1427.

[19] H. Zollinger, *Angew. Chem. Int. Ed. Engl.* **1978**, *17*, 141.

[20] C. Yang, Q. Jin, H. Zhang, J. Liao, J. Zhu, B. Yu, J. Deng, *Green Chem.* **2009**, *11*, 1401.
